# High‐Purity Functional Corneal Endothelial Cells From Human Induced Pluripotent Stem Cells via a Novel Wash‐Out Method

**DOI:** 10.1002/mco2.70650

**Published:** 2026-03-20

**Authors:** Eun‐Ah Ye, Changmin Kim, Minah Jeon, Yeji Yoon, Jiyoon Park, Ryun Hee Lee, Carson Yu, Ho Seok Chung, Jae Yong Kim, David Myung, Hun Lee

**Affiliations:** ^1^ Department of Ophthalmology Asan Medical Center University of Ulsan College of Medicine Seoul South Korea; ^2^ Department of Ophthalmology AMIST, Asan Medical Center University of Ulsan College of Medicine Seoul South Korea; ^3^ Department of Ophthalmology Brain Korea 21 Project University of Ulsan College of Medicine Seoul South Korea; ^4^ Department of Ophthalmology Spencer Center for Vision Research Byers Eye Institute At Stanford University Palo Alto California USA; ^5^ Department of Chemical Engineering Stanford University Stanford California USA; ^6^ Center For Cell Therapy Asan Medical Center Seoul South Korea

**Keywords:** animal model, cell therapy, clinical‐grade human induced pluripotent stem cells, corneal endothelial‐like cells, corneal endothelial dysfunction, single‐cell RNA sequencing

## Abstract

Corneal endothelial failure can cause blindness, with transplantation as the only treatment. Due to donor shortages, establishing robust methods for generating corneal endothelial‐like cells (CECs) from induced pluripotent stem cells (iPSCs) is critical. Differentiation protocols included iPSC‐to‐CEC induction with or without neural crest cell differentiation. CECs directly differentiated from iPSCs demonstrated robust expression of CEC‐specific markers and a hexagonal morphology. The wash‐out protocol is a novel, efficient, noncytotoxic method for removing undifferentiated iPSCs and obtaining CEC populations with high purity. Single‐cell sequencing data showed that iPSC–CECs with wash‐out were similar to human primary CECs. In vivo transplantation of iPSC–CECs into a corneal endothelial dysfunction (CED) rabbit model demonstrated their safety and therapeutic efficacy, with improved corneal transparency. Notable recovery of corneal clarity in the CED model, without graft rejection, highlights the in vitro and in vivo potential of iPSC–CECs as a powerful source for clinical therapy in patients with CED. This work establishes an effective stem cell‐based platform for producing corneal endothelium‐like cells with clinical‐grade quality, offering a scalable and regenerative alternative to conventional transplantation.

## Graphical abstract

iPSC‐derived corneal endothelial cells purified via a novel wash‐out method show high functional fidelity, resemble primary CECs, and restore corneal clarity in vivo, offering a scalable alternative to donor transplantation.

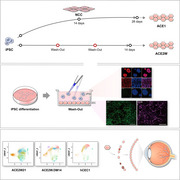

## 1 Introduction

Corneal endothelium is the innermost layer of the cornea, comprising a single layer of hexagonal cells interconnected by tight junctions, that plays a role in maintaining corneal transparency and functionality via pumping action to maintain appropriate hydration levels [1]. Corneal endothelial cells (CECs) are arrested in the G1 phase of the cell cycle and have a very limited capacity for proliferation; therefore, any significant cell loss cannot be naturally replenished [2].

Multiple factors can lead to corneal endothelial failure, including aging, trauma, ocular surgery, infection, and genetic or degenerative disorders such as Fuchs endothelial corneal dystrophy (FECD). Currently, nearly 300 million people over the age of 30 years are estimated to be affected by FECD [3]. Currently, corneal transplantation remains the only effective treatment for corneal endothelial dysfunction (CED). However, the availability of transplantable donor corneas remains critically low [4]. The high cost of transplantation and the need for specialized surgical skills further limit accessibility.

Endothelial keratoplasty techniques including Descemet's stripping automated endothelial keratoplasty, Descemet's stripping endothelial keratoplasty, and Descemet's membrane (DM) endothelial keratoplasty have significantly improved clinical outcomes compared with full‐thickness transplantation. These methods selectively replace only the diseased endothelium, reducing postoperative complications and recovery time. Nevertheless, the success of these techniques remains highly dependent on surgical expertise [5, 6], and donor shortage continues to be a major obstacle.

Induced pluripotent stem cells (iPSCs) offer a particularly promising and ethically favorable alternative for regenerative medicine [7]. However, only a few studies have focused on iPSCs and CECs [8, 9, 10, 11, 12]. Utilizing stem cell resources resolves the problems generated when using human donor corneas, including insufficient donor corneas, heterogeneous cell populations over time, and changes in CEC functionality and phenotype following the second passage [12, 13]. However, iPSC differentiation protocols frequently yield variable results between different cell lines, and the differentiation potential of individual iPSC lines can vary [14]. Therefore, using the clinical‐grade hiPSCs, we developed and utilized a novel method to generate high‐quality and high‐purity CEC population by washing undifferentiated stem cells out of the differentiated populations and demonstrated their therapeutic efficacy and safety in an animal model of CED.

## 2 Results

### 2.1 Characterization of CEC Induced From hiPSC: Direct Differentiation

**FIGURE 1:**
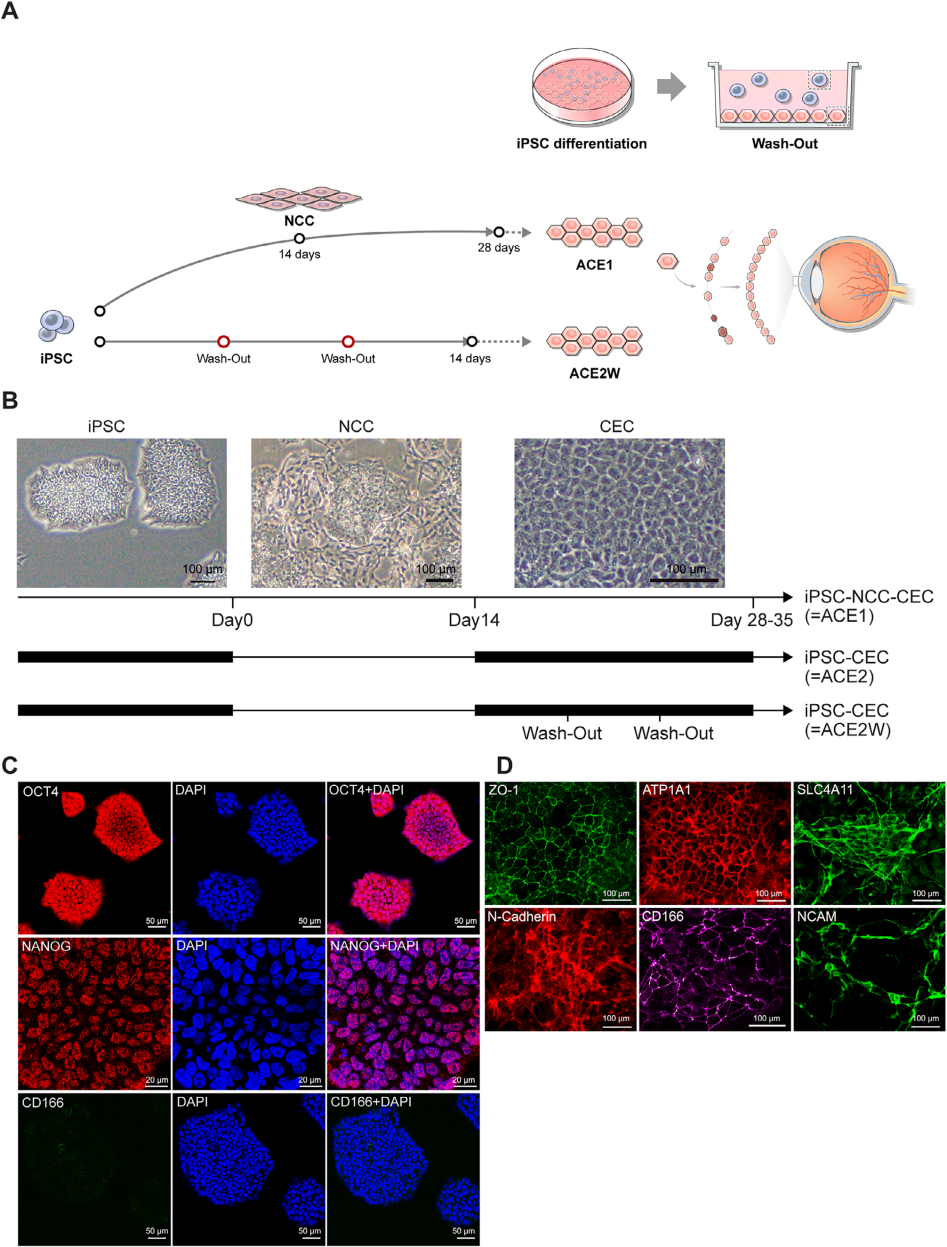
Characterization of corneal endothelial‐like cells (CECs) generated from induced pluripotent stem cells (iPSCs). (A) The wash‐out protocol enables more differentiated CECs to adhere at a faster rate to the VTN‐coated surface during the initial stage, while relatively immature cells with weaker attachment capacity are subsequently washed out and removed. A control culture dish without the wash‐out step is used for comparison of the initial cell seeding number. When approximately 50% of the seeded cells had attached to the culture surface, the remaining unattached cells were removed by aspirating the medium containing unattached cells, followed by a single gentle wash with PBS for less than 1 min. The culture was then replenished with fresh medium at the same volume routinely used for maintenance and returned to incubation. The wash‐out protocol was applied to the ACE2 cell population at each passage during the differentiation process and was performed once weekly upon reaching confluence, resulting in two wash‐out procedures during the 14‐day differentiation protocol and three during the 21‐day protocol. (B) Protocol for the differentiation of iPSCs into CECs. iPSCs were differentiated into CECs through either a neural crest cell (NCC) intermediate stage or directly, without the NCC stage. Phase‐contrast microscopy revealed CEC‐specific hexagonal morphology after the differentiation. Scale bars = 100 µm. (C) Immunocytochemistry for OCT4 and NANOG, iPSC markers, and CD166, a CEC marker, on iPSC colonies. No CD166‐IR was detected in the iPSC colonies. Scale bars = 50 µm (OCT4, CD166); scale bars = 20 µm (NANOG). (D) Immunocytochemistry for a group of CEC‐specific markers; zona occludens‐1 (ZO‐1; a tight‐junction protein), pump function protein Na^+^/K^+^ ATPase α1 (ATP1A1), SLC4A11, N‐CADHERIN, CD166, and NCAM on iPSC‐derived CECs. Scale bars = 100 µm.

iPSCs were differentiated into CECs either through a neural crest cell (NCC) stage (ACE1) or directly (ACE2), bypassing the NCC stage (Figure 1A,B). For direct differentiation of iPSCs into CECs (ACE2), the NCC step was omitted, and iPSCs were exposed to CEC‐differentiation medium in the subsequent weeks. We further developed a novel protocol to improve cell purity during CEC differentiation involving application of a wash‐out method (Figure 1A,B) that exploits the distinct adhesion properties of undifferentiated stem cells and mature CECs. This wash‐out protocol allows more differentiated CECs to adhere to a vitronectin (VTN)‐coated surface in the initial stage, whereas the remaining undifferentiated cell population, with relatively weaker attachment ability, is washed out and removed. We also tested and compared an alternative culture medium, CTS–DMEM, with endo‐SFM. iPSC–CECs cultured in this medium for 14 days were designated as ACE2W.DM14 (Table 1).

**TABLE 1:** Seven groups of cell populations used for single‐cell RNA sequencing.

Sample no.	Cell population	Days of differentiation	Wash‐out	Differentiation medium
1	ACE1	14	N	Human endothelial‐SFM
2	ACE2	14	N	
3	ACE2W14	14	Y	
4	ACE2W21	21	Y	
5	ACE2W.DM14	14	Y	CTS KnockOut DMEM/F‐12
6	hCEC1	—	—	DMEM/F‐12
7	hCEC2	—	—	DMEM/F‐12

(1) ACE1 (NCC‐mediated differentiation) cells were differentiated from iPSCs via the NCC intermediate stage. The differentiation period was 14 days, following a two‐step protocol (iPSC → NCC → CEC); (2) ACE2 (direct differentiation) cells were directly differentiated from iPSCs into CECs without passing through the NCC stage. Differentiation was performed for 14 days; (3) ACE2W14 (direct differentiation with wash‐out, 14 days) cells were directly differentiated from iPSCs for 14 days with an additional wash‐out step during the differentiation process. The term “W” denotes this wash‐out treatment; (4) ACE2W21 (direct differentiation with wash‐out, 21 days) cells were generated under the same conditions as ACE2W14, but with an extended differentiation period of 21 days. The notation “21” indicates the number of days in differentiation; (5) ACE2W.DM14 (direct differentiation with wash‐out, 14 days, AOF medium) cells were differentiated directly from iPSCs for 14 days with the wash‐out step applied, using CTS KnockOut DMEM/F‐12 as the basal medium. This formulation is animal‐origin‐free (AOF), distinguishing it from the other ACE populations that were cultured in human endothelial‐SFM medium; (6, 7) hCEC1 and hCEC2 (primary human CECs) from two independent human donors were maintained in DMEM/F‐12‐based culture medium, serving as physiological reference controls for the iPSC‐derived CEC populations.

As differentiation progressed, the cell morphology changed to display a CEC‐like hexagonal shape, starting around one week after initiating the CEC‐differentiation procedure. We performed immunocytochemistry (ICC) to characterize both iPSCs and CEC‐like cells using a panel of iPSC‐ and CEC‐specific markers. iPSCs showed immunostaining with antibodies against OCT4 and NANOG, but not CD166 (Figure 1C). After CEC differentiation, iPSC–CECs displayed robust expression of ZO‐1, ATP1A1, SLC4A11, N‐CADHERIN, CD166, and NCAM (Figure 1D). Both direct differentiation and iPSC–NCC–CEC methods resulted in comparable levels of marker expression in the populations, demonstrating successful induction of CECs from iPSCs using an efficient differentiation protocol.

### 2.2 Single‐Cell Analysis Revealed CEC Characteristics and the Superiority of iPSC–CECs

**FIGURE 2:**
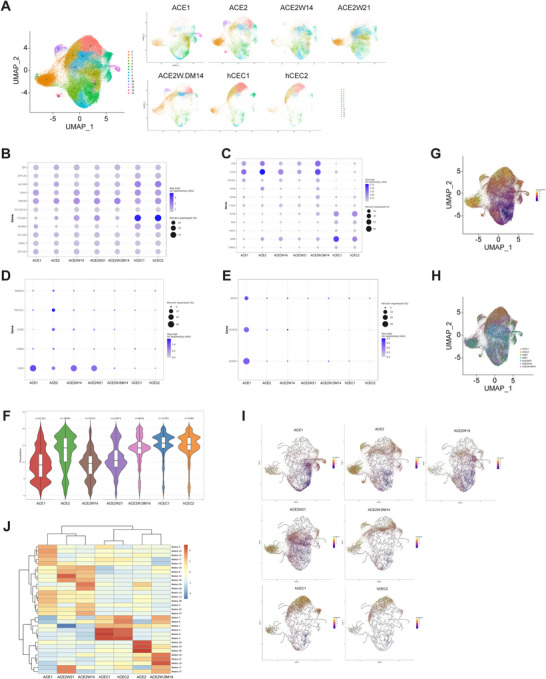
Single‐cell profiling of iPSC‐ and CEC‐specific genes across different populations of iPSC‐derived CECs. Single‐cell RNA sequencing was performed on seven different cell populations, including ACE1, ACE2, ACE2W14, ACE2W21, ACE2W.DM14, and primary human CECs isolated from two different donors. (A) UMAP DimPlot with gene cluster labels. UMAP DimPlot with gene cluster labels split by group. (B–E) Dot plots were generated to visualize the expression patterns of specific gene groups: (B) typical CEC marker genes, (C) low‐quality CEC‐associated genes, (D) pluripotency‐related iPSC genes, and (E) neural crest cell (NCC) genes. (F) Pseudotime distributions of each cell population are shown. The ACE2 groups gradually shifted toward the pseudotime region corresponding to hCECs, indicating a transition toward a more mature CEC phenotype. (G–J)Trajectory and pseudotime analysis were performed. (G) Trajectory graph with pseudotime: using the selected root state (Cluster 5 of the Seurat clusters), cells were assigned pseudotime values based on their projection onto the principal graph constructed by the algorithm. (H) Trajectory graph with sample: the distribution of cells from each experimental group is visualized along the inferred differentiation trajectory. (I) UMAP plots with pseudotime gradient: each group's single‐cell UMAP visualization is colored according to pseudotime, with the gradient changing from purple (early) to yellow (late) differentiation stages. (J) Heatmap of gene expression modules: clustering based on module‐level gene expression patterns derived from coexpression network analysis of single‐cell transcriptomes.

In total, seven RNA libraries from different samples were prepared using the Chromium Next GEM Single Cell 3p RNA Library v3.1 (10× Genomics), and 36,601 genes and 89,192 cells were analyzed. Fourteen gene clusters were identified and grouped from a total of seven cell population samples (Figure 2A and Table 1). Based on the cell cluster distribution in the uniform manifold approximation and projection (UMAP), the ACE2W.DM14 population showed the highest similarity to hCECs (Figure 2A). The expression profiles of specific gene groups were analyzed, including (Figure 2B) typical CEC marker genes (*TJP1*, *ATP1A1*, *ALCAM*, *CDH2*, *PRDX6*, *SLC25A11*, *COL8A1*, *NCAM1*, *SLC3A2*, *VDAC3*, *ATP1B1*); (Figure 2C) low‐quality CEC‐associated genes (*CD9*, *CD24*, *PROM1*, *DPP4*, *CD44*, *ITGA5*, *NT5E*, *ENG*, *VMO1*, *MME*, *THBS2*); (Figure 2D) pluripotency‐related iPSC genes (*NANOG*, *POU5F1*, *ESRG*, *CNMD*, *SOX2*); and (Figure 2E) NCC genes (*NGFR*, *HOXC8*, *HOXB7*). Gene expression was visualized by dot plots (Figure 2B–E) and UMAP analyses (Figure S1A). The majority of cells in the ACE1 and ACE2 populations exhibited strong expression of typical CEC markers (Figures 2B and S1B). For low‐quality CEC‐related genes, expression patterns varied among the genes and cell groups compared with hCECs (Figure 2C). For pluripotency‐related iPSC genes, the effect of the wash‐out method was evident: the ACE2 population contained several iPSC gene‐expressing cells, whereas very few were detected in the ACE2W populations (Figures 2D and S1C). Notably, the ACE2W.DM14 group contained the fewest cells expressing iPSC markers (Figure 2D). Unlike other populations, ACE1 cells expressed NCC‐associated markers (*NGFR*, *HOXC8*, *HOXB7*) (Figures 2E and S1D), which likely reflects the presence of residual, incompletely differentiated NCC‐derived stem cells, as ACE1 cells progress through an NCC stage during the differentiation protocol. Overall, the ACE populations exhibited comparable expression levels of typical CEC markers relative to hCECs, and the wash‐out method effectively removed undifferentiated stem cells, particularly in the ACE2W.DM14 population, which showed the greatest similarity to hCECs.

Pseudotime and trajectory analyses were performed to evaluate cellular maturation dynamics (Figure 2F–J). The ACE2 groups gradually shifted toward the pseudotime region corresponding to hCECs, indicating progression toward a more mature CEC phenotype. The ACE2 population (without wash‐out) exhibited greater heterogeneity compared with the wash‐out groups, whereas ACE2W21 showed more advanced CEC‐like characteristics than ACE2W14. Among all conditions, ACE2W.DM14 demonstrated the most mature and hCEC‐like transcriptional profile (Figure 2F). The trajectory graph with pseudotime shows how pseudotime values were assigned to each cell by projecting them onto the principal graph, using the selected root state (Figure 2G) and the trajectory graph categorized by sample visualizes how cells from each experimental group are distributed along the inferred differentiation pathway (Figure 2H). Together, these trajectory maps contextualize the subsequent UMAP‐based visualization of lineage progression. UMAP plots colored by pseudotime (Figure 2I) illustrate the differentiation trajectory, with gradients ranging from purple (early) to yellow (late) stages. Among the conditions tested in this study, the ACE2W.DM14 condition exhibited a relatively more advanced and stable CEC‐like state. Furthermore, heatmap analysis of gene expression modules revealed differential activation across groups, showing that ACE2 and ACE2W.DM14 shared module activation patterns most similar to those of hCECs (Figure 2J). Collectively, these results suggest that the ACE2W.DM14 population exhibits more mature and hCEC‐like transcriptional and functional characteristics compared with the other tested conditions.

**FIGURE 3:**
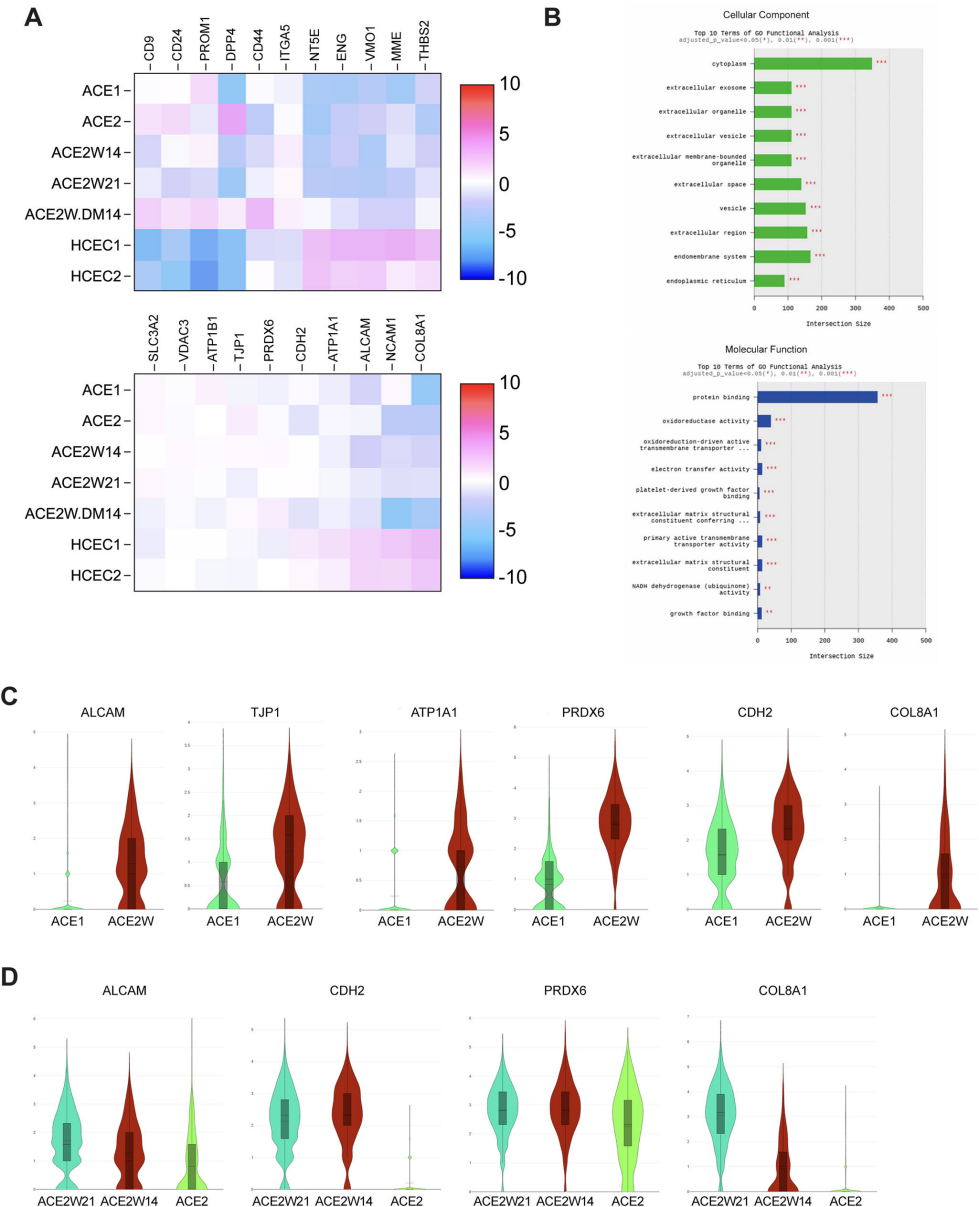
Gene expression features of ACE populations and functional annotation analysis. (A) Heatmap of typical CEC gene list. A group of CEC‐specific genes was compared for expression between different cell populations. The ACE2W groups showed higher levels of gene expression and improved profiles compared with those in the ACE2 population. Heatmap of low‐quality CEC markers. ACE populations displayed better quality overall compared with that of primary hCECs. (B) Functional annotation analysis for differentially expressed genes per cluster. The bar chart shows the number of genes enriched in each term. The enriched gene ontology (GO) terms in gene Cluster 10 of the ACE2W21 population are displayed. Notable similarities were found in many functions, including extracellular vesicle, focal adhesion, cell–substrate junction, mitochondrial protein‐containing complex, oxidoreductase activity, electron transfer activity, NADH dehydrogenase activity, inner mitochondrial membrane protein complex, growth factor binding, and collagen‐containing ECM, between the ACE2W21 and hCEC clusters. (C) Violin plots for CEC‐specific genes between ACE1 and ACE2 (wash‐out) groups. Both populations were iPSC‐derived CECs at differentiation Day 14. Expression levels of ALCAM, TJP1, ATP1A1, PRDX6, CDH2, and COL8A1 genes, as well as the number of cells expressing these genes, were significantly increased in ACE2 compared with those in ACE1. *y*‐axis = Log2 expression. (D) Among ACE2 populations, the wash‐out groups displayed distinct elevation of CEC‐specific genes compared with that in the no‐wash‐out group at D14. The expression of ALCAM and COL8A1 were further increased at D21 as the differentiation period progressed. *y*‐axis = Log2 expression.

Overall, similar patterns of CEC gene expression were observed between iPSC–CECs and primary hCECs. A group of CEC‐specific genes was identified and reported [15, 16, 17], and our heatmap displayed relative expression levels between samples. Expression levels of CEC genes among each population were not significantly different, but hCECs displayed relatively stronger expression of some genes. The cell populations, including hCECs, exhibited varying levels of gene expression—stronger or weaker—depending on the gene type (Figure 3A). For fibroblastic‐ or low‐quality CEC markers (Figure 3A, upper panel), ACE populations exhibited a better expression profile compared with that of primary hCECs. ACE2W21 showed the best characteristics in terms of CEC‐specific genes. The ACE2W.DM14 group showed robust expression of typical CEC markers, comparable to that of other ACE2 populations (Figure 3A, bottom panel). For the gene set of low‐quality CEC markers, ACE2W.DM14 displayed slightly higher levels of some genes compared with those in other ACE2 groups (Figure 3A, upper panel).

### 2.3 Functional Similarity Between iPSC–CECs and Primary hCECs

The same enriched gene ontology (GO) terms as in primary hCECs (Cluster 0 and 2) were observed in gene cluster 10 of the ACE2W21 population (Figure 3B). Notable similarities were found in many functions, including extracellular vesicle, focal adhesion, cell–substrate junction, mitochondrial protein‐containing complex, electron transfer activity, oxidoreductase activity, NADH dehydrogenase activity, inner mitochondrial membrane protein complex, growth factor binding, and collagen‐containing ECM, between ACE2W21 and hCEC clusters. These elevated gene expression levels in the population indicate an enhanced capacity for metabolic activity, tissue maintenance, cellular signaling, and repair processes. ACE2W cells have the potential to exert therapeutic efficacy in pathological conditions by adapting to stress, repairing tissue damage, and maintaining corneal transparency and integrity. Overall, ACE2W21 population showed the most functional similarities to hCECs.

The ACE2 (Day 14, no‐wash‐out) population (Cluster 14) showed enrichment of GO terms related to cell adhesion molecule binding, tight junction, cadherin binding, intracellular membrane‐bounded organelle, ATP binding, purine ribonucleoside triphosphate binding, enzyme binding, and GTPase regulator activity (Figure S2). This could indicate increased metabolic activity, protein synthesis, and cellular organization, particularly in proliferating cells. It suggests active involvement in signaling pathways, especially those regulating cell growth and differentiation. ACE2W14 (Cluster 13) showed enriched GO terms related to cytoskeleton, spindle, mitotic spindle, nuclear chromosome, histone binding, chromatin binding, actin binding, mRNA binding, enzyme binding, and nucleoplasm. This could indicate increased cytoskeletal reorganization, cellular remodeling and cell responses to active cell division, growth, and differentiation. ACE2 and ACE2W14 showed broadly similar patterns in gene‐expression regulation and enzyme activity (Figure S2). However, their functional characteristics diverged. ACE2 cells were enriched for pathways related to cell adhesion and metabolic regulation, emphasizing structural stability and organellar function. In contrast, ACE2W14 cells were predominantly associated with cell proliferation, cytoskeletal dynamics, and genomic regulation. ACE1 population (Cluster 12) exhibited markedly higher expression of neural development or neural crest‐related genes (*NGFR, KALRN, MAP1B, TUBB2B, TUBB3, CRABP1, HES6, DCC, STMN2, EBF1, HOXC8*), which is likely reflective of the neural crest–like state during differentiation. Collectively, the ACE2W21 population exhibited the highest similarity to primary hCECs in terms of CEC marker expression and functional annotation analysis, compared with that of all other groups.

Between the ACE1 and ACE2W14 groups at differentiation Day 14, the expression levels of CEC‐specific genes (*ALCAM, TJP1, ATP1A1, PRDX6, CDH2*, and *COL8A1*), as well as the number of cells expressing these genes, were significantly higher in the ACE2W14 group compared with those in ACE1 (Figure 3C). Moreover, among ACE2 populations, the wash‐out groups exhibited distinct elevation of CEC‐specific genes compared with those in the no‐wash‐out group at D14. *ALCAM* and *COL8A1* expression were further increased at D21 as the differentiation period progressed (Figure 3D).

### 2.4 Differentiation Into Functional, High‐Purity CECs: Efficacy of the Wash‐Out Method

**FIGURE 4:**
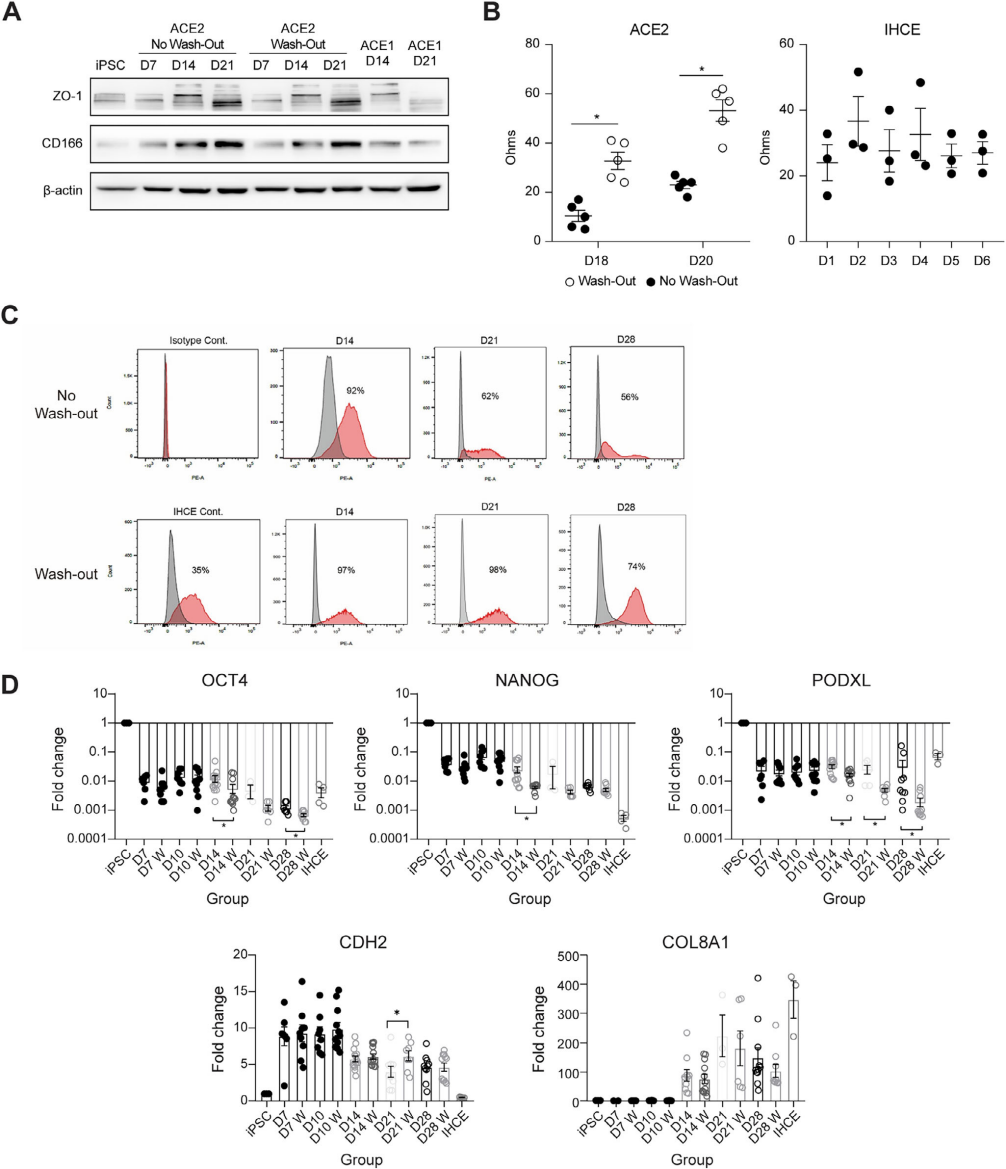
Molecular and functional analysis of iPSC‐derived CECs. (A) Western blot analysis of CEC marker expression comparing wash‐out and no‐wash‐out conditions. (B) Transepithelial/endothelial electrical resistance (TEER) measurements indicating the integrity of junctional formation. TEER comparison between wash‐out and no‐wash‐out groups, with the wash‐out condition showing significantly increased values at Day 18 and Day 20. IHCE was used for comparison (*N* = 5 for ACE2 groups; *N* = 3 for IHCE). (C) Flow cytometry analysis. The CD166 antibody was used to identify high‐quality CECs, and wash‐out groups showed notably higher levels of CD166 positivity compared with that in the no‐wash‐out populations. The highest expression of the marker (98%) was found in the wash‐out group at D21. (D) Gene expression levels of iPSC (*OCT4, NANOG, PODXL*) and CEC (*CDH2, COL8A1*) markers were quantified by RT‐qPCR. Significant differences were found between the wash‐out and no‐wash‐out groups after D14 for iPSC markers. CDH2 expression levels were significantly elevated in the wash‐out group at D21. IHCE was used for comparison (*N* = 3; *p < 0.05). Data are presented as mean ± SEM. *Abbreviation*: IHCE = immortalized human corneal endothelial cells.

Protein expression of CEC markers (ZO‐1, CD166, N‐CADHERIN, ATP1A1, and SLC4A11) was demonstrated by western blot (Figures 4A and S3). We also examined the endothelial functionality of iPSC–CECs by performing transepithelial/endothelial electrical resistance (TEER) measurements. Measurements of the ohmic resistance value were taken 2 days after cell seeding and repeated for the following 2 days. The comparison between the wash‐out and no‐wash‐out groups further confirmed the impact of the wash‐out procedure, with TEER values showing significant increases at both Day 18 and Day 20 following wash‐out (Figure 4B). TEER values for ACE2W.DM14, which were cultured and measured in a different medium composition (AOF medium supplemented with clinically qualified fetal bovine serum), are presented separately in Figure S4. Immortalized human corneal endothelial cells (IHCE) were used for comparison. The results indicated good barrier integrity of tight junctions in iPSC–CECs (ACE2) when compared with that in IHCE.

We then performed flow cytometry for quantitative measurement using CD166, a marker associated with high‐quality CECs. By removing undifferentiated cells from the iPSC–CEC population, we achieved a high level of cell purity with 98% of CD166‐positive cells at Day 21. In contrast, the no‐wash‐out group showed relatively lower percentages of CD166‐positive cells over time (Figure 4C). Additional flow‐cytometry data using ATP1A1 demonstrated the wash‐out‐mediated improvement in CEC purity (88.1 vs. 80.9%, wash‐out vs. no‐wash‐out) (Figure S5). To further validate the impact of the wash‐out method on CEC purity and quality, ICC was performed to assess marker expression together with cellular morphology (Figures S6 and S7). Across all examined markers—CD166, ATP1A1, ZO‐1, SLC4A11, PITX2, and N‐CADHERIN—the wash‐out group consistently exhibited higher CEC purity and superior overall quality compared with the no‐wash‐out group.

The gene expression levels of iPSC markers (*OCT4*, *NANOG*, *PODXL*) were significantly reduced to nearly undetectable levels in the wash‐out group after D14. Additionally, *CDH2* expression was found to be significantly higher in the wash‐out group at D21 (Figure 4D). These findings confirm that the wash‐out method is a safe, straightforward, and highly effective approach for removing undifferentiated stem cells and improving CEC purity during differentiation.

### 2.5 Therapeutic Efficacy of iPSC–CEC Transplantation

**FIGURE 5:**
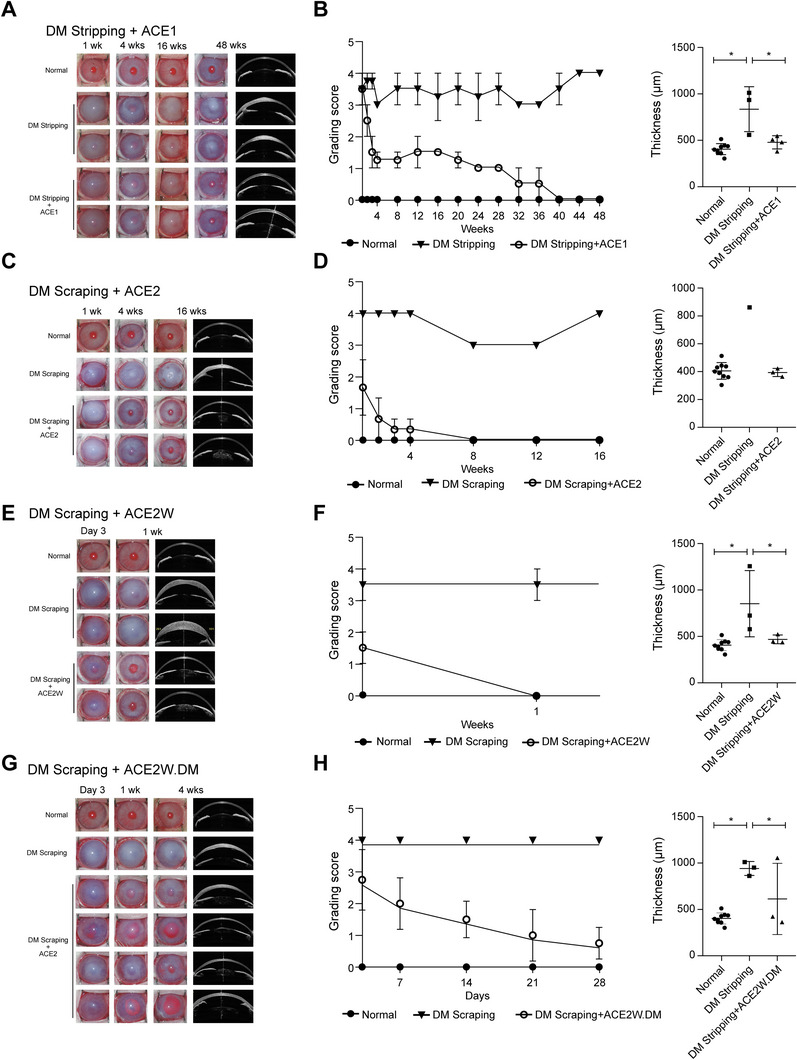
Therapeutic efficacy of iPSC‐derived CECs in a rabbit CED model. Ocular observations and OCT imaging at different time points for the endothelial scraping and DM stripping models. (A) ACE1 injection in a DM stripping model (*N* = 4), (C) ACE2 injection in a DM scraping model (*N* = 3), (E) ACE2W injection in a DM scraping model (*N* = 3), and (G) ACE2W.DM injection in a DM scraping model (*N* = 3). Compared with the nontransplanted control, corneal transparency was significantly improved over time in the transplanted groups. Corneal thickness was measured at the last time point using AS‐OCT images. Recovery from corneal edema was observed in each transplanted group (B, D, F, and H). Corneal recovery time in the DM stripping model (A) was longer than in the scraping models (C, E, and G). Faster recovery of corneal opacity was observed in the ACE2W transplant group (E) compared with that in the ACE2 group (C) (*p < 0.05). Data are presented as mean ± SEM.

We developed an animal model of CED by stripping off DM along with the endothelium or by scraping off the endothelial cells. iPSC–CECs were then injected into the anterior chamber (AC) of these models, and ocular observations were conducted from 1–48 weeks posttransplantation (PT). Anterior segment optical coherence tomography (AS‐OCT) was performed to accurately assess changes in the corneal structure and thickness. We observed that CEC transplantation resulted in noticeable recovery over time for all transplant groups examined (Figure 5).

For the DM stripping model, ACE1 cells were injected and recovery effects were observed up to 48 weeks PT, compared with those in the untreated control (Figure 5A). The cell transplantation group showed gradual recovery of corneal clarity through 48 weeks after treatment, whereas untreated control corneas remained unchanged throughout the 48 weeks PT (Figure 5B). For the DM scraping model, either ACE2 (no‐wash‐out) or ACE2W (wash‐out) cells were transplanted. Both cell groups were injected at Day 21 of differentiation. Following ACE2 injection (Figure 5C,D), recovery of corneal transparency was evident starting from 4 weeks PT compared with that in the untreated control. In the DM scraping group with ACE2W injection, noticeable improvements in corneal opacity were observed as early as 1 week PT (Figure 5E,F). After ACE2W.DM injection, recovery effects were also evident as early as 1 week PT, although there was variation between individuals (Figure 5G,H). Therapeutic effects of CEC injection on corneal edema were evidenced based on AS‐OCT data demonstrating that corneal swelling was resolved and thickness returned to levels comparable to that of normal corneas for all transplant groups. These results strongly indicate that our iPSC–CEC transplantation strategy successfully rescued the cornea from CED.

### 2.6 Detection of Transplanted CECs in the Host Eyes

**FIGURE 6:**
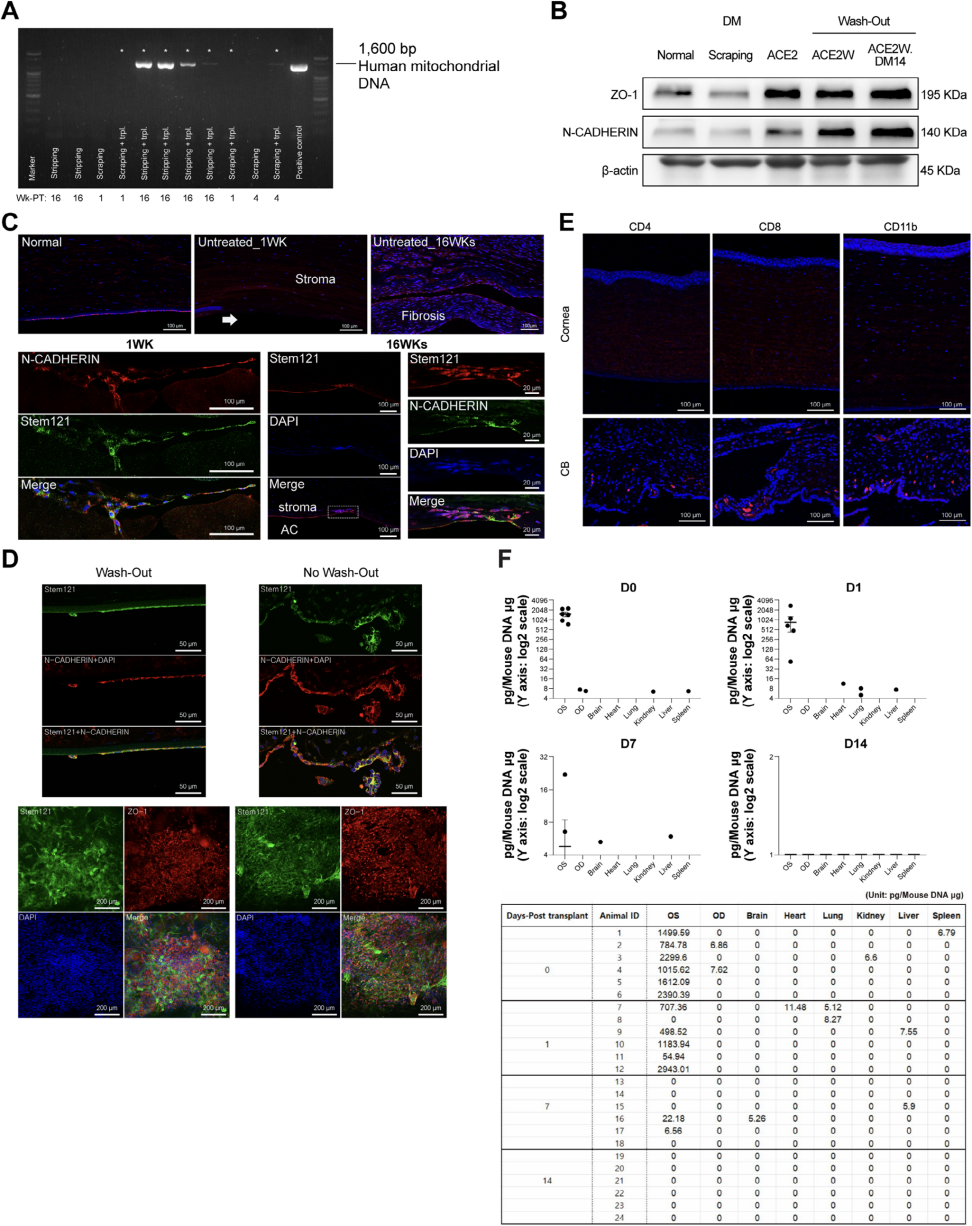
Survival of transplanted iPSC‐derived CECs in the host anterior chamber without immune activation. (A) Transplanted cells in host eyes were detected using PCR for human mitochondrial DNA. Mitochondrial DNA bands were observed in the cornea samples at 1, 4, and 16 weeks PT in both the DM‐scraping or ‐stripping groups. Asterisks (*) indicate individuals that received cell transplantation. (B) Western blot results showing bands against ZO‐1 and N‐CADHERIN in the cornea at 2 weeks PT. (C) Immunohistochemistry for the detection of a human‐specific marker (STEM121) and CEC marker (N‐CADHERIN) in the corneas at 1 and 16 weeks PT. Double‐labeling of STEM121 and N‐CADHERIN, indicating the survival of transplanted iPSC–CECs (ACE2W21), was observed along the endothelium in the rabbit eyes. Scale bars = 100 µm. (D) At 2 weeks posttransplantation, surviving transplanted cells were detected in both wash‐out and no‐wash‐out groups. The wash‐out group showed improved adhesion and a more continuous endothelial monolayer (top, scale bars = 50 µm). Whole‐mount staining confirmed surviving ACE2 cells (STEM121^+^/ZO‐1^+^) in both groups (bottom, scale bars = 200 µm). Overall cell survival was comparable between groups. (E) No immunoreactivity for T cells (CD4 and CD8) or macrophages/neutrophils (CD11b) was found in the transplanted groups at any of the time points examined. Representative images of ACE2 transplantation at 16 weeks posttransplantation are shown. Scale bars = 100 µm. (F) Biodistribution of transplanted cells in a mouse model of cryoinjury. PCR for human Alu DNA was performed to detect transplanted human iPSC–CECs in the mouse model of CED. The transplantation groups included: (1) Day 0 of cell injection (*N* = 6), (2) Day 1 after cell transplantation (*N* = 6), (3) Day 7 after transplantation (*N* = 6), and (4) Day 14 after transplantation (*N* = 6). OS (left eye, received transplantation), OD (contralateral eye, no transplantation), brain, kidney, liver, lung, heart, and spleen were collected at 0, 1, 7, and 14 days PT (*y*‐axis = Log2 scale).

We evaluated the treated corneas for the presence of transplanted iPSC–CECs. After harvesting corneal tissues, DNA was isolated and amplified for human mitochondrial genes. Human mitochondrial gene expression was detected in the host corneas at different time points, including 1, 4, and 16 weeks PT (Figure 6A). Not all host corneas contained human mitochondria, which may be attributed to interindividual variation. The presence of transplanted human iPSC–CECs (ACE1) was also confirmed by assessing human nuclear antigen in the treated corneas. Western blot analysis revealed detection of human nuclear proteins in the host corneas at 48 weeks PT (Figure S8). For the ACE2 groups, a comparison between wash‐out and no‐wash‐out conditions was performed (Figure 6B). Relative to the scraping control, all ACE‐transplanted groups showed increased ZO‐1 and N‐CADHERIN expression. Among them, the ACE2W21 group exhibited a significantly higher level of ZO‐1 compared with the scraping control group (Figure S9). We performed histological examinations using antibodies against CEC‐ and human‐specific markers (Figure 6C). Double labeling with the human‐specific marker stem121 and N‐CADHERIN demonstrated the survival of transplanted human iPSC–CECs (ACE2W21) in the rabbit corneal endothelium at 1 and 16 weeks PT. To further evaluate the efficacy of the wash‐out procedure, we compared the transplantation outcomes between the wash‐out and no‐wash‐out groups (Figure 6D). At 2 weeks PT, surviving transplanted cells—identified as double positive for STEM121 and ZO‐1—were detected in the host corneal endothelium in both groups. The wash‐out group exhibited better adhesion and a more continuous endothelial monolayer compared with the no‐wash‐out group (top panel). Whole‐mount staining also confirmed the presence of surviving ACE2 cells in both groups (bottom panel). Overall, no substantial differences in the survival of transplanted cells were observed between the groups at 2 weeks PT.

To assess this, we immunostained T cells and macrophages using antibodies against CD4, CD8, and CD11b. The results of ACE2 transplantation at 16 weeks PT are presented (Figure 6E). Analyses were performed for both the wash‐out and no‐wash‐out ACE2 groups at 1, 2, and 16 weeks PT. Neither group showed evidence of immune‐cell activation in the corneal region, which is consistent with the absence of immunoreactivity for the examined immune cell markers in the representative images. Ciliary body and iris tissues were used as positive controls to confirm the appropriate immunoreactivity of each antibody. Overall, transplanted iPSC–CECs in the CED model were found to have survived and induced significant regeneration of the corneal endothelium without significant immune rejection or inflammatory responses.

### 2.7 Biodistribution of Transplanted Cells in Vivo

PCR for human Alu DNA was performed to detect transplanted human iPSC–CECs in a mouse model of CED. In total, seven major organs or tissues were assessed, including the left eye (receiving transplantation), right eye (contralateral eye), brain, kidney, liver, lung, heart, and spleen, collected at 0, 1, 7, and 14 days PT (Figure 6F). A large amount of human DNA was found in the left eye at Day 0, with a slight decrease over time, showing some variations between individuals. At Day 7, only a small amount of human DNA was detected in the left eye. Other organs, such as the lung, spleen, kidney, liver, heart, and brain, showed very low amounts of human DNA from Days 0 to 7 PT. No human DNA was detected at Day 14 in any of the tissues examined.

We also performed cell injection using CECs derived from a different source of hiPSCs, which were of nonclinical grade (Figure S10), in the CED rabbit model. Long‐term results after transplanting iPSC‐derived CECs showed a gradual recovery of corneal transparency over 48 weeks following transplantation. Biodistribution analysis using qPCR for the human Alu gene demonstrated that the transplanted cells were present only in the treated eye (2.664 pg) and not in other major organs (brain, kidney, liver, lung, and heart) or the contralateral eye at 48 weeks PT.

## 3 Discussion

In this study, direct differentiation bypassing the NCC stage appeared to yield better results in terms of differentiation efficiency and cellular functionality. Transcriptomic data also showed higher functional similarity between ACE2 and hCECs than between ACE1 and hCECs. This may be attributed to the higher flexibility of iPSCs in giving rise to various cellular lineages and fates, compared with that of cells at the NCC stage, which retain limited differentiation potential and are likely to differentiate into non‐CEC populations, such as neural lineages. ACE2W displayed even higher functional similarity to hCECs than that of ACE2. Moreover, in ACE2W, the functionality of mature CECs was more pronounced at D21 than at D14. ACE2W cells are highly active and robust, capable of efficient intercellular communication while maintaining structural integrity, and exhibiting enhanced capacity for metabolic activity. The wash‐out method is a simple, efficient, and nondamaging approach to purify the CEC population, relying on the distinct cell–matrix adhesion capacities between iPSC–CECs and undifferentiated stem cells [18]. Because the wash‐out method simply involves gently removing the culture medium from the flask, it is completely noncytotoxic to the cells. It requires no additional staining or specialized equipment—which can induce cellular stress, decrease viability, and increase cost—making the method simple, cost effective, and highly efficient for eliminating undifferentiated stem cells and markedly improving CEC purity during differentiation. Given these advantages and the absence of any chemical or mechanical manipulation, the wash‐out method represents a practical and clinically relevant strategy for generating high‐purity CECs.

Primary CECs in cultures in vitro tend to show morphological change to a spindle shape at later passages, characteristic of endothelial‐to‐mesenchymal transition [17]. Further, CEC‐specific markers are lacking and identification of CECs remains a challenge. It is thus necessary to use a group of well‐known markers to best characterize the cells. Most commonly used markers, such as Na/K ATPase, ZO‐1, COL8, and SLC4 subtypes, are frequently employed to identify CECs [15, 17, 19]. Additionally, ALCAM is considered a marker for high‐quality, mature human CECs and is not expressed in cells with altered morphology. In contrast, *CD44, VMO1, MME*, and *THBS2* are expressed in low‐quality CECs and are absent in high‐quality CEC cultures [16]. Furthermore, CD24–, CD26–, CD44–^/dull^, CD105–^/dull^, CD133–, and CD166^+^ cells are considered mature, differentiated hCECs under cultured conditions [20]. ACE2W populations exhibited high levels of ALCAM expression after 14 days of differentiation, along with good morphology. Regarding low‐quality CEC marker profiles, the ACE2W population at Day 21 displayed the best characteristics of CECs among the iPSC–CEC groups tested.

Our therapeutic strategy of CEC transplantation resulted in a successful outcome, rescuing the corneas from CED. In the DM scraping model, eyes treated with ACE2W injection exhibited faster recovery, with improvements observed as early as 1 week PT, compared with those in the ACE2 group, where recovery was evident at 4 weeks PT. Both in vitro and in vivo results demonstrated the enhanced efficacy of iPSC–CECs generated using our novel protocol in the CED model. Host eyes maintained immune homeostasis stably, with or without immunosuppression, following xenograft injection. This suggests that corneal immune privilege was well‐maintained, without significantly stimulating the host immune system after transplantation. Importantly, we investigated both the stripping‐DM‐off and scraping‐endothelium‐off models for inducing CED. Considering clinical applications that may involve severe cases of CED, we assessed the therapeutic efficacy using a model involving stripping of the DM. A diameter of 8–9 mm was completely removed [21]. The rescue effects of iPSC–CEC transplantation were remarkable over time, while nontreated control eyes remained opaque and swollen for the long‐term period (up to 48 weeks PT). Many of the corneas that were stripped showed development of stromal fibrosis. Posterior corneal fibrosis may resolve slowly and gradually, occurring up to 1 year posttreatment (ACE1) [22]. iPSC–CEC transplantation may play a potential role in resolving fibrosis more quickly than that in the untreated group. Transplantation of iPSC–CECs may also alleviate edema, thereby restoring corneal curvature and reducing mechanical stress, which are inducing factors of fibrosis [23]. Fibrosis can influence the survival of transplants [24], in addition to immune rejection. Therefore, developing an antifibrosis strategy may be an effective approach to prevent the accumulation of myofibroblasts in the CED model.

The persistence of transplanted iPSC–CECs is another crucial aspect to be evaluated. We found that the transplanted human material could still be detected in the host rabbit eye 1 year PT. However, other organs and tissues, such as the brain, liver, lung, kidney, and heart, did not show any detectable levels of human Alu. Additionally, when assessing the biodistribution of the transplanted iPSC–CECs at various time points following cell injection in a mouse model of CED, other major organs, such as lung, spleen, kidney, liver, heart, and brain did not show any detectable human DNA from Day 14 PT. From the rabbit and mouse models of CED, we did not observe any severe side effects or signs of tumorigenicity at any of the time points examined, for up to 48 weeks. Our previous study [11], using hiPSCs derived from fibroblasts, demonstrated that transplantation of iPSC–CECs did not result in tumor formation in vivo, confirming the safety of the procedure. These results provide additional convincing evidence regarding the use of iPSCs and their therapeutic efficacy in treating ophthalmological disorders [25].

We observed remaining human mitochondrial DNA in the host corneas up to 48 weeks PT at the time points when no detectable human cells were observed by immunohistochemistry using a human nuclear antigen antibody. Along with molecular evidence, we observed in vivo functional recovery of the transplanted eyes over an extended period following transplantation. Healthy mitochondria can be transferred to adjacent cells and tissues, exerting effects on cell survival, energy production, and maintaining cell function [26, 27]. Mitochondrial DNA is especially susceptible to oxidative stress, and mutations in mitochondrial DNA have been reported in patients with FECD [28]. The possibility of a therapeutic approach involving intercellular mitochondrial transfer from mesenchymal stem cells to damaged human CECs and 661 W cells has been reported, showing improved outcomes in the recipient cellular function [29]. Prolonged therapeutic efficacy through the transfer of iPSC–CEC‐derived mitochondria might be a potential molecular mechanism underlying corneal endothelial regeneration and recovery in our CED model. Additionally, evaluating therapeutic efficacy in nonhuman primate models may provide more supporting evidence and represent our next step toward clinical research.

One of the challenges in treating dysfunctional corneal endothelium with single‐cell suspensions is the successful delivery and adhesion of transplanted cells onto the interior surface of the cornea [30]. Various approaches have been explored, including engineering biomaterials to generate biocompatible and biodegradable sheets for CEC delivery [31, 32, 33]. One previous study assessed the effects of monkey‐CEC sheet transplantation into the eyes of cynomolgus monkey after endothelial scraping. Transplant survival was observed on Day 7 following CEC sheet transplantation, and the effects on corneal edema were observed from 2 weeks to 6 months PT [33]. These strategies may enhance the adhesion of transplanted cells to the host corneal surface and promote their prolonged retention. Using bioengineered scaffolds in combination with iPSC–CECs could be an effective strategy to achieve more efficient cell delivery to the target site. Further material improvements are needed to optimize delivery performance into the AC, ensure good transparency, and enhance sturdiness for handling during surgery.

In conclusion, our study provides notable outcomes with a novel method to generate high‐purity, functional CECs from clinical‐grade iPSCs. ACE2 wash‐out groups—particularly ACE2W21 and ACE2W.DM14—most closely resemble primary hCECs and exhibit greater therapeutic potency than the no‐wash‐out ACE2 group and ACE1. The wash‐out procedure enhances CEC purity during differentiation by effectively removing residual undifferentiated stem cells, thereby reducing the potential risk of tumorigenicity—a critical consideration in the development of cell‐based therapeutics. Furthermore, among the groups evaluated in this study, ACE2W.DM14 appears to be the most optimal CEC population among all groups, as it is generated under AOF conditions and demonstrates the highest CEC purity. Therefore, it represents a promising candidate for potential clinical applications, offering advantages in terms of safety, consistency, and therapeutic efficacy.

### 3.1 Limitations of the Study

Several limitations should be considered. In addition to challenges related to (1) the successful delivery and adhesion of transplanted CECs as a single‐cell suspension, and (2) the need for combined antifibrosis strategies mentioned earlier, (3) the long‐term stability and functionality of the generated CECs require further evaluation in diverse in vivo environments. Although no tumors or ectopic engraftment were detected by Alu qPCR and histological analyses up to 48 weeks, the possibility of very low‐frequency residual cells or late‐onset events cannot be entirely ruled out given the sample size and detection limits. In addition, because ACE1 was primarily evaluated in a DM‐stripping model, whereas ACE2 and ACE2W were assessed in DM‐scraping models, direct comparisons of relative efficacy across these conditions are inherently limited and should be interpreted with caution. Future studies are needed to assess the potential of iPSC–CECs in a broader range of animal models and human clinical settings to address these limitations and fully evaluate the safety, efficacy, and long‐term outcomes of iPSC–CEC‐based therapies for corneal endothelial diseases.

## 4 Materials and Methods

### 4.1 Study Design

We used New Zealand White rabbits (*N* = 33) to generate a CED model. Our study examined only male animals to eliminate potential biological variability associated with female animals. The sample size or number of biological replicates is specified in each figure legend. Clinical grading to assess corneal transparency was performed in a blinded manner, while all other analyses were conducted in a nonblinded fashion. Details of each experimental model and procedure are described below.

### 4.2 hiPSC Differentiation Into CECs

Clinical‐grade hiPSCs derived from cord blood were provided by our collaborator, Yipcell Inc. The hiPSCs were derived and banked under GMP‐compliant conditions, while subsequent thawing, maintenance, and experimental procedures were conducted in a non‐GMP research laboratory setting. The hiPSCs were cultured in essential 8 medium with supplements (Gibco, Carlsbad, CA) on culture plates coated with VTN (Life Technologies, Carlsbad, CA). Two different protocols were used to generate CECs: the first method involved differentiating iPSCs into CECs via the NCC stage (ACE1), while the second method entailed direct differentiation of iPSCs into CECs without the NCC step (ACE2).

For NCC differentiation, iPSCs were plated onto a VTN‐coated surface. When the iPSCs reached 30–40% confluency, medium was replaced with a series of NCC induction media. One day following differentiation, medium was changed to E8 medium supplemented with 10 µM retinoic acid and 5 µM CHIR99021 (Selleckchem, Houston, TX). The following day (Day 2), the medium was replaced with E8 medium supplemented with 5 µM retinoic acid (Sigma–Aldrich, St. Louis, MO), 10 µM SB431542, 5 µM CHIR99021, and 500 nM LDN193189 (Selleckchem). On Day 3, the medium was switched to E8 medium with 5 µM retinoic acid, 10 µM SB431542, 5 µM CHIR99021, and 100 nM BGJ398 (Selleckchem). On Day 4, the culture medium was replaced with the PSC Neural Induction Medium Kit (Cat# A1647801; Gibco), and the cells were subsequently fed daily for an additional 10 days. During this extended culture period, subculturing was performed upon reaching confluence at a seeding density of 10,000 cells/cm^2^. No additional small molecules or growth factors were added during the 10‐day culture period.

### 4.3 A Cryoinjury Mouse Model of CED

C57BL/6J mice were anesthetized via subcutaneous injection of 30 mg/kg tiletamine/zolazepam (Zoletil 50, Virbac) and 10 mg/kg xylazine hydrochloride (Rompun, Bayer). Cryoinjury was induced using a stainless steel probe with a 1.5 mm diameter, pre‐cooled in liquid nitrogen at −80°C for 3 min in the left eye of each mouse. The probe was applied to the left cornea for 3 s, forming an ice ball on the corneal surface. After each application, the probe was recooled in liquid nitrogen for an additional 30 s. Each mouse underwent three cryoinjury cycles.

A total of 24 mice received injections of iPSC–CECs (ACE2W21) at a concentration of 54,054 cells per eye. Intracameral injection was performed using a Hamilton syringe (80308–701SN, 10 µL; 33‐gauge needle, 20 mm length, 4‐point needle tip). A volume of 1–2 µL of cell suspension was slowly injected into the AC. Following injection, the animals were maintained in a prone position with the transplanted eye facing downward for 3 h to facilitate cell attachment.

Tissue or organ samples (eye, brain, kidney, liver, lung, heart, and spleen) were collected at different time points: 0, 1, 7, and 14 days PT (*n* = 6 for each time point).

### 4.4 Differentiation Into CECs

iPSC‐derived NCCs or iPSCs were plated onto 35‐mm plates coated with VTN. CEC differentiation medium was prepared by supplementing the medium with 1% insulin–transferrin–selenium (ITS; Life Technologies), 0.02 mg/mL 2‐phosphate ascorbic acid, 1 µM SB431542 (Selleckchem), 0.2 mg/mL CaCl_2_ (Sigma–Aldrich), 2.5 µM H‐1152 (Tocris, Abington), 10 ng/mL hEGF, and 10 µM fasudil to human endothelial‐SFM medium (Gibco). Medium was changed every other day for 2–4 weeks. Differentiated cells were passaged upon reaching approximately 90% confluence.

An additional culture medium was tested, including the human endothelial‐SFM medium. CTS KnockOut DMEM/F‐12 (Thermo Fisher Scientific, Waltham, MA) with 5% FBS (Hyclone; Cat# SV30207), 1% ITS, 0.2 mg/mL CaCl_2_, 0.02 mg/mL 2‐phosphate ascorbic acid, 1 µM SB431542, 2.5 µM H‐1152, 10 ng/mL hEGF, and 10 µM fasudil, to induce CEC differentiation for 14 days. CTS–DMEM is an AOF product.

### 4.5 Cell Populations and Differentiation Protocols

Seven different CEC populations were analyzed, including iPSC‐derived CECs generated through distinct differentiation strategies and primary human CECs. The characteristics of each group are summarized (Table 1).

### 4.6 Human Primary CEC Culture

Human primary CECs were isolated from two different donor corneas (Eversight, Chicago, IL), expanded in vitro, and subcultured until Passage 2. Cells were plated onto VTN‐coated culture plates and maintained in DMEM/F12 medium (Gibco) supplemented with 5% FBS, 1% ITS, 10 µM Y27632, 0.02 mg/mL ascorbic acid, 0.02 mg/mL CaCl_2_, 10 ng/mL hEGF, and 1% antibiotic–antimycotic (Gibco). The medium was replaced every 2–3 days. Cells were used for RNA sequencing and immunostaining as a control group. The information for the donor corneas and cultured CECs is as follows: Donor #1: age 41 years, female, White, Caucasian, CEC Passage 2; Donor #2: age 27 years, female, White, Caucasian, CEC Passage 2.

### 4.7 Cell Transplantation Into the CED Model

New Zealand white rabbits (1.8–2.2 kg, *N* = 33) were accommodated in standard cages under optimal environmental control. The room temperature was maintained at 24°C with a 12‐h light/dark cycle. The CED model was generated, and the surgical procedures were performed by an experienced physician (HL). Triamcinolone was administered by a single subconjunctival injection 3 days prior to transplantation. No systemic immunosuppression was used. Animals were anesthetized with 5 mg/kg tiletamine/zolazepam (Zoletil 50, Virbac) and 2 mg/kg xylazine hydrochloride (Rompun, Bayer) by intramuscular injection. A viscoelastic agent was injected into the AC, followed by either endothelial scraping or DM stripping [33, 34, 35, 36, 37]. The DM was scored and stripped off from the posterior stroma in a circular pattern using a reverse Sinskey hook [36]. The stripped area measured 8.9 ± 0.2 mm in diameter, and viscoelastic agent was thoroughly removed. Subsequently, cells (1 × 10^6^ iPSC–CECs in 150 µL of PBS supplemented with 100 µM of Fasudil) were injected into the AC through a paracentesis incision and monitored for up to 16 weeks. Cells within comparable passage number ranges were used for all transplantation experiments. The animal groups were as follows: (1) ACE1 injection into a DM stripping model (*N* = 4), (2) ACE2 injection into a DM scraping model (*N* = 3), (3) ACE2W injection into a DM scraping model (*N* = 3), and (4) ACE2W.DM14 injection into a DM scraping model (*N* = 3). Animals in the nontransplantation control group received a PBS injection following endothelial scraping or DM stripping. Following injection, the animals were maintained in a prone position with the transplanted eye facing downward for 3 h to facilitate cell attachment. Two rabbits injected with iPSC–CECs (36A; nonclinical grade) were observed for up to 48 weeks. Corneal opacity was graded using a slit‐lamp microscope on a scale of 0–4 (0: clear; 1: slight turbidity with visible iris texture; 2: moderate turbidity with obscured iris texture; 3: marked turbidity with a faintly visible pupil; 4: severe turbidity with no visible pupil) [38, 39]. Corneal tissues were collected at 1, 4, 16, and 48 weeks after cell transplantation for further analysis. All procedures conformed to the Association for Research in Vision and Ophthalmology (ARVO) Statement for the Use of Animals in Ophthalmic and Vision Research (ARVO Animal Policy). This study was approved by the Institutional Review Board (2022‐0753). Experiments involving live vertebrates were conducted in strict accordance with relevant national and international guidelines for animal care and use, as mandated by the IACUC of Asan Medical Center (2022‐12‐119).

### 4.8 Real‐Time Quantitative PCR

Total RNA from iPSCs and iPSC‐derived CECs was extracted using TRIzol reagent (Invitrogen, Carlsbad, CA). Complementary DNA was synthesized using a cDNA synthesis kit (Enzynomics, Daejeon) and RT‐qPCR was performed using a Power SYBR Green PCR Master Mix (Applied Biosystems, CA) on a QuantStudio 3 System (Applied Biosystems). Gene expression levels of iPSC markers (*NANOG*, *OCT4*, and *PODXL*), and CEC‐associated markers (*CDH2*, *COL8A1*) were quantified at different time points of culture (iPSC and CEC induction at 7, 14, 21, and 28 days). *GAPDH* was utilized as the internal control. The relative expression of genes was calculated based on the formula 2^(−ΔΔCt)^.   ΔΔCt values are ΔCt_exp._ − ΔCt_cont._ The primer sequences are summarized in Table 2.

**TABLE 2:** Primer sequences used. (A) Sequences used for RT‐qPCR. (B) Sequences used for human mitochondrial DNA detection. (C) Sequences used for human Alu detection.

Gene (A)	Primer sequence (5′–3′)
*GAPDH*_F	TCC AGA ACA TCA TCC CTG CC
*GAPDH*_R	GCC TGC TTC ACC ACC TTC TT
*CDH2*_F	GAC CAG GAC TAT GAC TTG AGC C
*CDH2*_R	AGC TGT GGG GTC ATT GTC AG
*COL8A1* 3F	GCC TCT CTC CCT GAT CTT ACG
*COL8A1* 3R	GCA GCA CAG CCA TCA CAT TT
*POU5F1*_F	TTT TGG TAC CCC AGG CTA TG
*POU5F1*_R	GCA GGC ACC TCA GTT TGA AT
*NANOG*_F	ACC TTG GCT GCC GTC TCT GG
*NANOG*_R	AGC AAA GCC TCC CAA TCC CAA ACA
*PODXL*_F	AAC TGG GCA AAG TGT GAG GA
*PODXL*_R	ACT TAT CTT GGG CCG GGT TG
Gene (B)	Primer sequence (5′–3′)
Human mito 3F	GCC TTC CCC CGT AAA TGA TA
Human mito 3R	CTT CTG TGG AAC GAG GGT TT
Human mito 5F	GCC GAC CGT TGA CTA TTC TC
Human mito 5R	GGG GGC ATC CAT ATA GTC AC
Gene (C)	Primer sequence (5′–3′)
Alu_F	TGG TGG CTC TCT CCT GTA AT
Alu_R	GAT CTC GGC TCA CTG CAA C
Reporter (probe)	FAM‐TGAGGCAGGAGAATCGCTTGAACC‐MGB

### 4.9 Single‐Cell RNA Sequencing

Single‐cell RNA sequencing was performed using the 10× Genomics platform. In total, seven different sample RNA libraries were prepared using the Chromium Next GEM Single Cell 3p RNA library v3.1, and profiling of 3′ digital gene expression of 5000–20,000 individual cells per sample was performed (Table 1). In total, 36,601 genes and 89,192 cells were analyzed. The samples were sequenced and processed using Cell Ranger 7.0.1. Cell population analysis was performed using the Seurat4.3.0 R package. Functional annotation for differentially expressed genes per cluster was also performed. Single‐cell trajectory construction and pseudotime analysis were performed using Monocle3 (v0.1.2) to identify cellular transitions between states.

### 4.10 TEER Measurement

iPSC‐derived CECs were seeded onto 24‐well cell culture inserts (0.4 µm; Merck, Rahway, NJ) at a density of 150,000 cells/cm^2^. Then, 300 and 1000 µL of culture medium were added into the upper and lower chamber, respectively. Cells were incubated for 2 days after seeding to allow the formation of an endothelial monolayer. The ohmic resistance value was recorded at different time points over the course of the experiment using the EVOM3 system (World Precision Instruments, Sarasota, FL), and the value was calculated by subtracting the resistance of the control insert chamber (without cells, medium only).

### 4.11 Human Alu PCR for Biodistribution Analysis

To investigate the biodistribution of transplanted human cells, PCR analysis was performed (Theranovis, Seoul) using a human Alu primer at 0, 1, 7, and 14 days PT. A cryoinjury mouse model was used to assess the transplant biodistribution [40]. In rabbit CED model, PCR analysis was performed using a human Alu primer at 48 weeks PT. DNA was extracted from each tissue sample (eye globe, brain, kidney, liver, lung, heart, and spleen) using AccuPrepGenomic DNA Extraction kit (Bioneer; K‐3032). RT‐qPCR was conducted using TaqMan Fast Advanced Master Mix (Thermo Fisher Scientific; 4444557) with 100 ng of DNA in a total volume of 20 µL. The reaction was performed as described in the kit instructions with annealing and extension at 60°C for 60 s. The primer sequence used is shown in Table 2. The data were analyzed by comparing the Ct values of the samples with the standard curve to quantify human DNA.

### 4.12 Immunocytochemistry

iPSC‐derived CECs were fixed in 4% paraformaldehyde for 30 min at room temperature, washed with PBS, and blocked in PBST (0.5% Triton X‐100 in PBS) containing 5% normal serum and 0.3% Triton X‐100. Cells were then incubated with the primary antibody in 1% bovine serum albumin (Sigma–Aldrich) in PBST overnight at 4°C, followed by incubation with goat anti‐mouse IgG Alexa Fluor 488‐conjugated and goat anti‐rabbit IgG Cy3‐conjugated secondary antibody (1:500; Jackson Immunoresearch, West Grove, PA). The antibodies used included human anti‐ZO‐1 (1:250; Life Technologies), SLC4A11 (1:250; Novus, Centennial, CO), N‐CADHERIN (1:200; Cell Signaling Technology, Danvers, MA), Na^+^/K^+^ ATPase α1 (1:200; Santa Cruz Biotechnology, Dallas, TX), CD166 (1:500; BD Biosciences, Franklin Lakes, NJ), PITX2 (100 µg/mL; Abnova, Taipei), and NCAM (1:200; R&D Systems, Minneapolis, MN). Nuclei were counterstained with DAPI (Sigma–Aldrich) for 10 min, and fluorescence signals were detected with a laser‐scanning confocal microscope (Olympus, Tokyo).

### 4.13 Western Blot Analysis

Cells were collected and lysed using RIPA buffer (EPIS BIO) supplemented with phosphatase and protease inhibitors (Sigma–Aldrich). Proteins were separated on 10% SDS‐polyacrylamide gels and transferred to PVDF membranes (Sigma–Aldrich) using a Trans‐Blot Cell system (Bio‐Rad, Richmond, CA). Membranes were blocked with 3% BSA (Sigma–Aldrich) in PBST to prevent nonspecific binding. Membranes were incubated overnight with antibodies against ZO‐1 (1:1000), SLC4A11 (1:250; Novus), N‐CADHERIN (1:200; Cell Signaling Technology), Na^+^/K^+^ ATPase α1 (1:200; Santa Cruz Biotechnology), human nuclear antigen (1:200; Abcam), and β‐actin (1:1,000; Cell Signaling Technology). Membrane was washed and incubated with an HRP‐conjugated anti‐mouse secondary antibody (GeneTex, Hsinchu) at a 1:10,000 dilution. Protein signals were visualized using a Gel DocTM XR+ imaging system (Bio‐Rad) according to the manufacturer's instructions. Beta‐actin was used to normalize the signal intensity of protein bands.

### 4.14 Flow Cytometry Analysis

iPSC‐derived CECs were harvested as pellets on differentiation Days 14, 21, and 28 and were further processed for cell staining. The resuspended cells were stained for the CEC marker CD166 in staining buffer FBS (Cat# 554656; BD Biosciences, San Jose, CA). An isotype control antibody was used as a negative control for staining. Antibody cocktail (10 µL) was added at 4°C for 10 min. The cells were then washed twice by adding 1 mL of flow cytometry buffer and centrifuging at 300×*g* for 10 min. The cells were then analyzed using a Becton Dickinson FACSCanto I flow cytometer and the flow cytometry plots were analyzed using FACSDiva software. Antibodies used were as follows: PE mouse anti‐human CD166 (Cat# 559263), PE mouse IgG1, κ Isotype control (Cat# 555749) (BD Biosciences), alpha 1 Sodium Potassium ATPase/ATP1A1 Antibody (C464.6) (Cat# sc‐21712 FITC; Santa Cruz Biotechnology), and FITC Mouse IgG1, κ Isotype Control (Cat# 555748; BD Biosciences). The staining procedure using the ATP1A1 antibody involved fixation and permeabilization steps.

### 4.15 Histological Examinations

Corneal tissues were fixed in 3.7% formaldehyde and subsequently embedded in paraffin. The paraffin blocks were sectioned into 4‐µm thick slices. Paraffin‐embedded sections were processed and stained with hematoxylin and eosin. Immunohistochemical analysis was performed by incubation with primary antibodies against: N‐CADHERIN (1:200; Cell Signaling), ZO‐1 (1:150), STEM121 (1:500; Takara), CD4 (1:1000; Novus), CD11b (1:100; Abcam), and CD8 (1:1000; Abcam). The secondary antibodies included the corresponding Alexa‐488 or ‐Cy3‐labeled antibodies (1:500; Abcam). Cell nuclei were counterstained with DAPI. Imaging was performed using a LSM710 confocal microscope (Carl Zeiss Meditec, Jena).

### 4.16 PCR Detection of Human Mitochondrial DNA

Total DNA was extracted from enucleated eyes 3 weeks after cell transplantation using a PicoPure DNA Extraction Kit (Life Technologies). PCR was then performed using NICSROgene BN‐Taq DNA Polymerase (Bionics, Seoul) with 200 ng of DNA in a total volume of 30 µL. The reaction was performed as described in the kit instructions with annealing at 58°C for 20 s.

### 4.17 Quantification and Statistical Analysis

Statistical analyses were conducted using the GraphPad Prism software (GraphPad, La Jolla, CA). Data are presented as mean ±  SEM. For comparisons involving three or more groups, one‐way or two‐way ANOVA with Bonferroni's multiple comparison test was conducted. Differences between two groups were evaluated using two‐tailed Student's *t*‐tests. Results with *p* values below 0.05 were considered statistically significant. For western blots, a representative blot was presented.

## Author Contributions

Conceptualization: EY and HL. Methodology: EY and HL. Investigation: CK, MJ, YY, JP, RL, and CY. Visualization: EY and CK. Funding acquisition: EY and HL. Project administration: EY. Supervision: HL. Writing – original draft: EY and HL. Writing – review and editing: EY, HC, JK, DM, and HL. All authors have read and approved the final manuscript.

## Funding Information

This work was supported by a grant of the Korea Health Technology R&D Project through the Korea Health Industry Development Institute (KHIDI), funded by the Ministry of Health & Welfare, Republic of Korea (grant number: RS‐2024‐00438366).

## Ethics Statement

All procedures conformed to the ARVO Statement for the Use of Animals in Ophthalmic and Vision Research (ARVO Animal Policy). This study was approved by the Institutional Review Board (IRB: 2022‐0753) at Asan Medical Center. Experiments on live vertebrates were conducted in strict accordance with the relevant national and international guidelines regarding animal handling as mandated by the Institutional Animal Care and Use Committee (IACUC) of the University of Ulsan College of Medicine (Seoul, Korea). The committee reviewed and approved our animal study protocol (2022‐12‐119).

## Conflicts of Interest

The authors declare no conflicts of interest.

## Supporting information




**Supporting Figure 1**: Uniform manifold approximation and projection (UMAP) analyses. (A) UMAP DimPlot with gene cluster labels. UMAP DimPlot with gene cluster labels split by group. (B) UMAP showing typical CEC‐specific genes (*TJP1*, *CDH2*, *ATP1A1*, *ALCAM*, *PRDX6*, *SLC25A11*) in ACE populations. (C) UMAP showing iPSC genes (*NANOG*, *POU5F1*, *ESRG*, *CNMD*). UMI counts are shown on a linear scale. (D) UMAP showing neural crest cell‐associated markers (*NGFR*, *HOXC8*, *HOXB7*).
**Supporting Figure 2**: Functional annotation analysis for the gene clusters corresponding to each cell population.
**Supporting Figure 3**: Western blot analysis of (A) ACE1 and ACE2 populations prior to implementation of the wash‐out method, and (B) comparison of wash‐out versus no‐wash‐out groups at Days 14 and 21 for selected CEC markers.
**Supporting Figure 4**: Transepithelial/endothelial electrical resistance (TEER) measurements of the ACE2W.DM population (differentiation Days 13–14).
**Supporting Figure 5**: Flow cytometry for ATP1A1 expression in the wash‐out and no‐wash‐out ACE2 populations at differentiation Day 21.
**Supporting Figure 6**: Immunocytochemistry (ICC) analysis of CEC markers in the wash‐out and no‐wash‐out ACE2 populations at differentiation Day 21.
**Supporting Figure 7**: Immunocytochemistry (ICC) analysis of CEC markers in the ACE2W.DM population at differentiation Day 14.
**Supporting Figure 8**: Western blot analysis of corneal tissue with ACE1 transplantation at 48 weeks posttransplant.
**Supporting Figure 9**: Western blot analysis of corneal tissue transplanted with different ACE2 populations at 2 weeks posttransplant. Quantitative data corresponding to Figure 6B are presented as graphs (*p < 0.05).
**Supporting Figure 10**: Biodistribution of transplanted iPSC‐derived CECs in a rabbit CED model.
**Supporting Table 1**: Key resources table.

## Data Availability

Further information and requests for resources and reagents should be directed to and will be fulfilled by the lead contact, Hun Lee (yhun777@amc.seoul.kr). The lead contact can provide information, including microscopy data, required to reanalyze the data reported in this paper upon request.
