# Supplementary material for: High‐Purity Functional Corneal Endothelial Cells From Human Induced Pluripotent Stem Cells via a Novel Wash‐Out Method
Source: MedComm (2020). 2026 Mar 20;7(4):e70650. doi: 10.1002/mco2.70650 (PMC13042470; doi:10.1002/mco2.70650)
Supplement: Supplementary file 1 — Supporting Figure 1: Uniform manifold approximation and projection (UMAP) analyses. (A) UMAP DimPlot with gene cluster labels. UMAP DimPlot with gene cluster labels split by group. (B) UMAP showing typical CEC‐specific genes (TJP1, CDH2, ATP1A1, ALCAM, PRDX6, SLC25A11) in ACE populations. (C) UMAP showing iPSC genes (NANOG, POU5F1, ESRG, CNMD). UMI counts are shown on a linear scale. (D) UMAP showing neural crest cell‐associated markers (NGFR, HOXC8, HOXB7). Supporting Figure 2: Functional annotation analysis for the gene clusters corresponding to each cell population. Supporting Figure 3: Western blot analysis of (A) ACE1 and ACE2 populations prior to implementation of the wash‐out method, and (B) comparison of wash‐out versus no‐wash‐out groups at Days 14 and 21 for selected CEC markers. Supporting Figure 4: Transepithelial/endothelial electrical resistance (TEER) measurements of the ACE2W.DM population (differentiation Days 13–14). Supporting Figure 5: Flow cytometry for ATP1A1 expression in the wash‐out and no‐wash‐out ACE2 populations at differentiation Day 21. Supporting Figure 6: Immunocytochemistry (ICC) analysis of CEC markers in the wash‐out and no‐wash‐out ACE2 populations at differentiation Day 21. Supporting Figure 7: Immunocytochemistry (ICC) analysis of CEC markers in the ACE2W.DM population at differentiation Day 14. Supporting Figure 8: Western blot analysis of corneal tissue with ACE1 transplantation at 48 weeks posttransplant. Supporting Figure 9: Western blot analysis of corneal tissue transplanted with different ACE2 populations at 2 weeks posttransplant. Quantitative data corresponding to Figure 6B are presented as graphs (*p < 0.05). Supporting Figure 10: Biodistribution of transplanted iPSC‐derived CECs in a rabbit CED model. Supporting Table 1: Key resources table. [file MCO2-7-e70650-s001.docx]

# Supplementary Information

**Title: High-Purity Functional Corneal Endothelial Cells from Human Induced Pluripotent Stem Cells via a Novel Wash-Out Method**

Eun-Ah Ye^1^, Changmin Kim^1,2,3^, Minah Jeon^1^, Yeji Yoon^1,2,3^, Jiyoon Park^1,2,3^, Ryun Hee Lee^1,2,3^, Carson Yu^1^, Ho Seok Chung^1^, Jae Yong Kim^1^, David Myung^4,5^, and Hun Lee^1,2,3,6^*

^1^Department of Ophthalmology, Asan Medical Center, University of Ulsan College of Medicine, Seoul, South Korea

^2^Department of Ophthalmology, AMIST, Asan Medical Center, University of Ulsan College of Medicine, Seoul, South Korea

^3^Department of Ophthalmology, Brain Korea 21 project, University of Ulsan College of Medicine, Seoul, South Korea

^4^Department of Ophthalmology, Spencer Center for Vision Research, Byers Eye Institute at Stanford University, Palo Alto, CA 94304, USA

^5^Department of Chemical Engineering, Stanford University, Stanford, CA 94305, USA

^6^Center for Cell Therapy, Asan Medical Center, Seoul, South Korea

*Correspondence and Lead contact: yhun777@ amc.seoul.kr; yhun777@gmail.com

This file contains supplementary figures (S1–S10) and a table (S1).

**
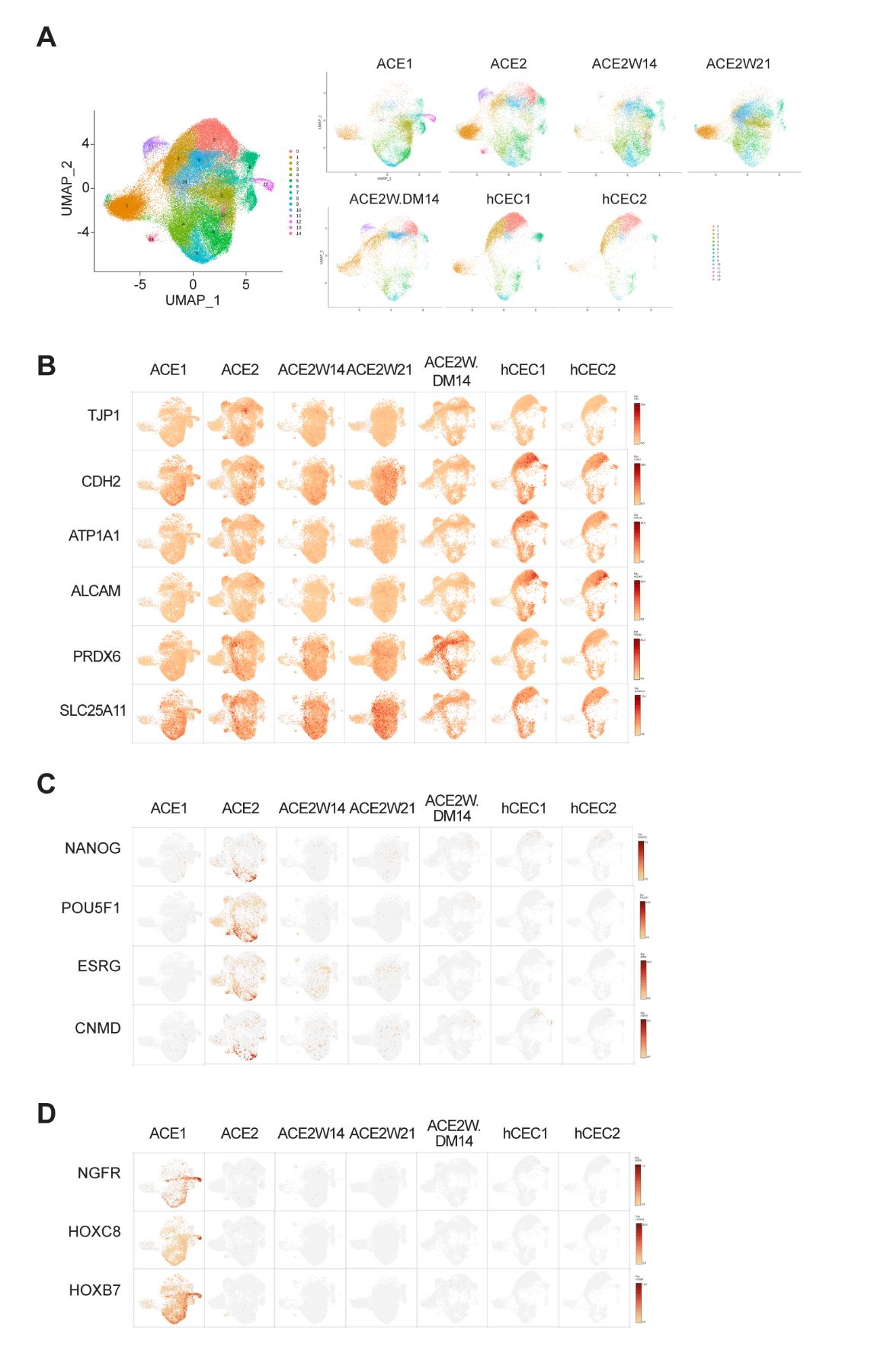
**

**Figure S1**. Uniform Manifold Approximation and Projection (UMAP) analyses. (A) UMAP DimPlot with gene cluster labels. UMAP DimPlot with gene cluster labels split by group. (B) UMAP showing typical CEC-specific genes (*TJP1, CDH2, ATP1A1, ALCAM, PRDX6, SLC25A11*) in ACE populations. (C) UMAP showing iPSC genes (*NANOG, POU5F1, ESRG, CNMD*). UMI counts are shown on a linear scale. (D) UMAP showing neural crest cell-associated markers (*NGFR, HOXC8, HOXB7*).


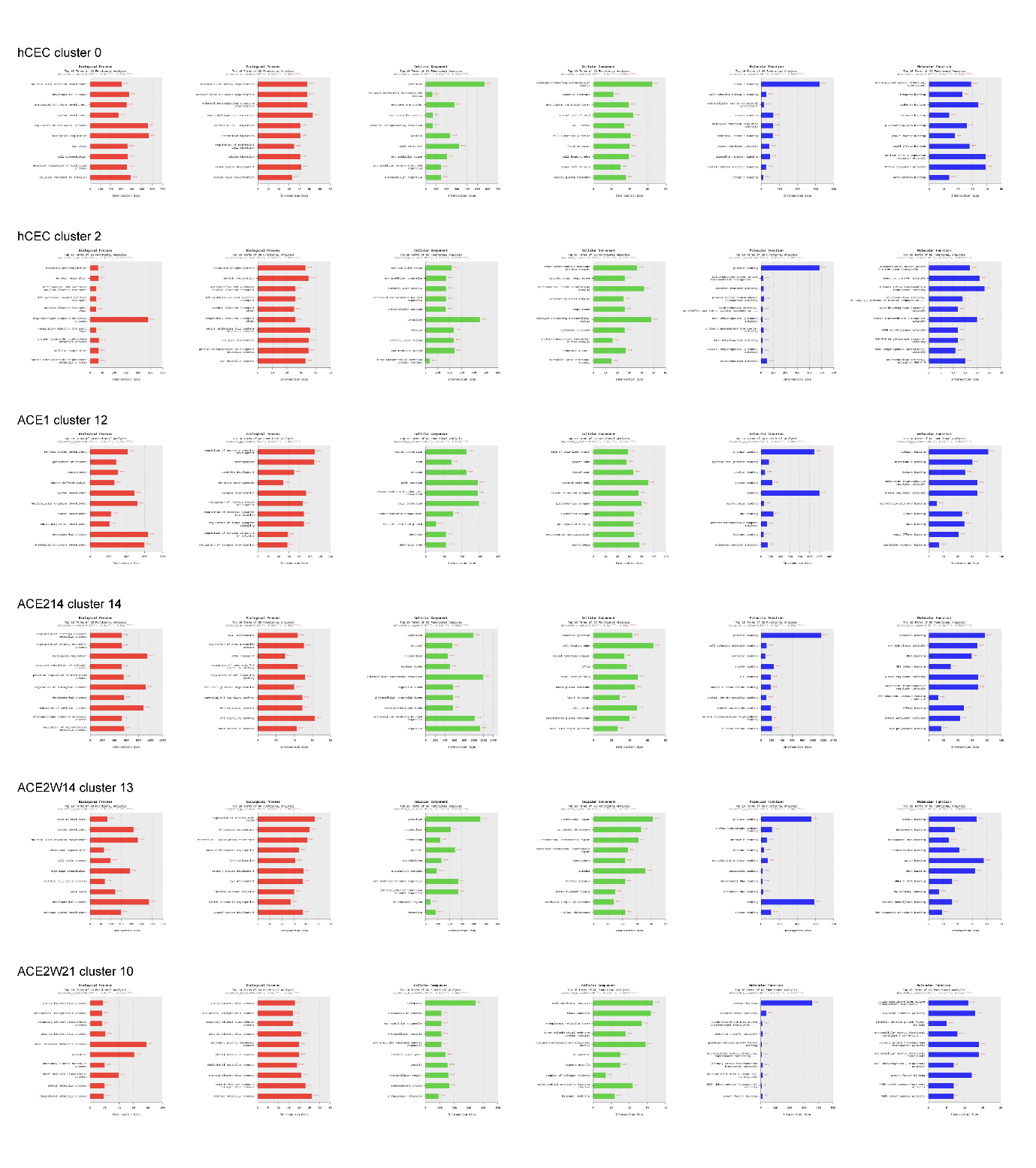


**Figure S2**. Functional annotation analysis for the gene clusters corresponding to each cell population.

**
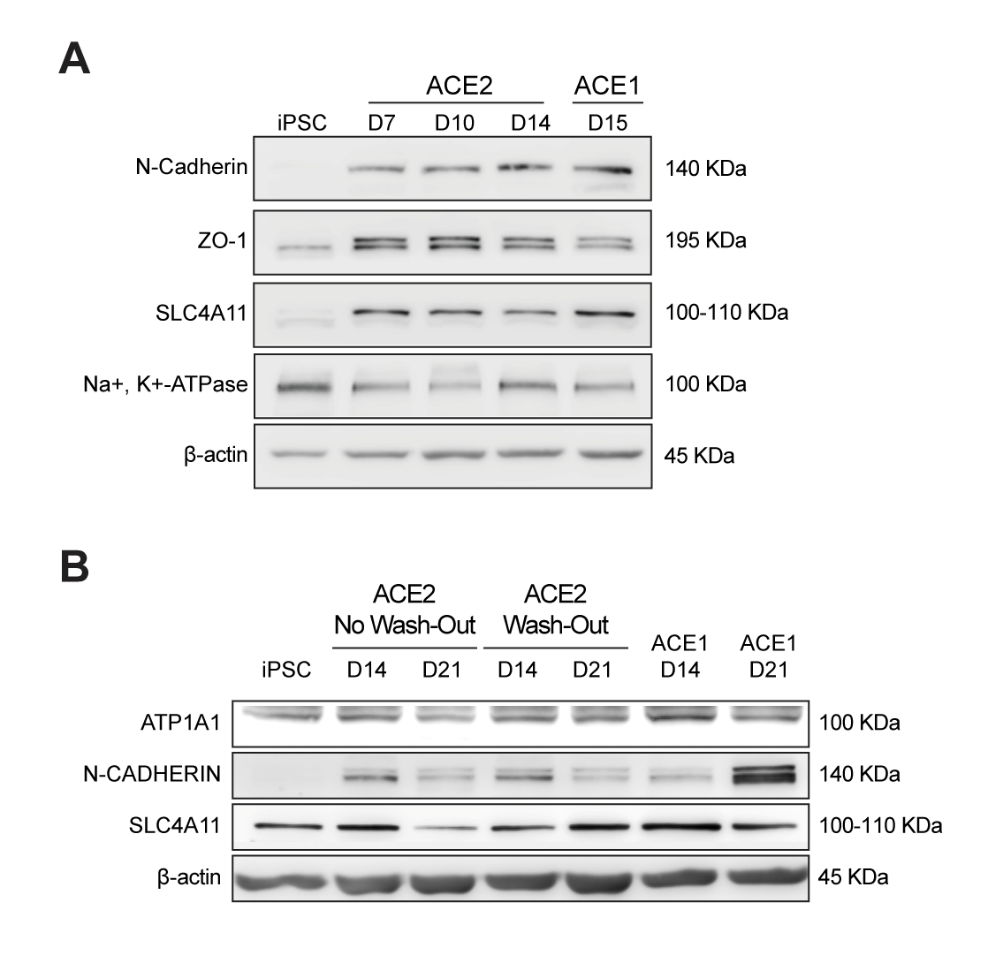
**

**Figure S3**. Western blot analysis of (A) ACE1 and ACE2 populations prior to implementation of the wash-out method, and (B) comparison of wash-out versus no-wash-out groups at days 14 and 21 for selected CEC markers.


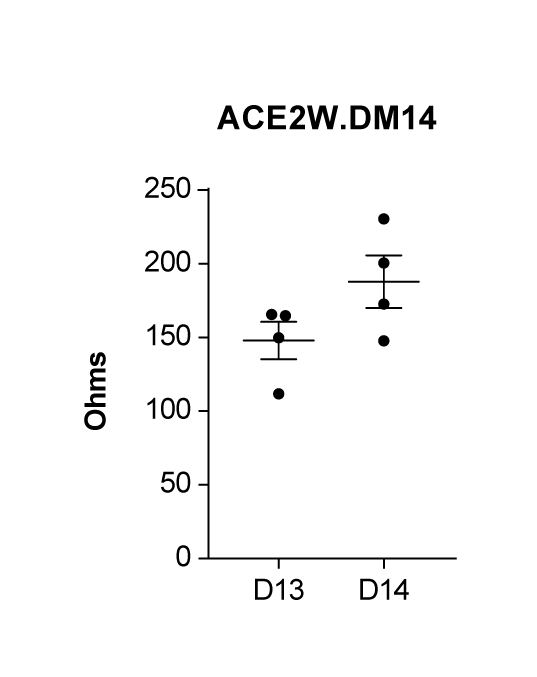


**Figure S4.** Transepithelial/endothelial electrical resistance (TEER) measurements of the ACE2W.DM population (differentiation days 13–14).


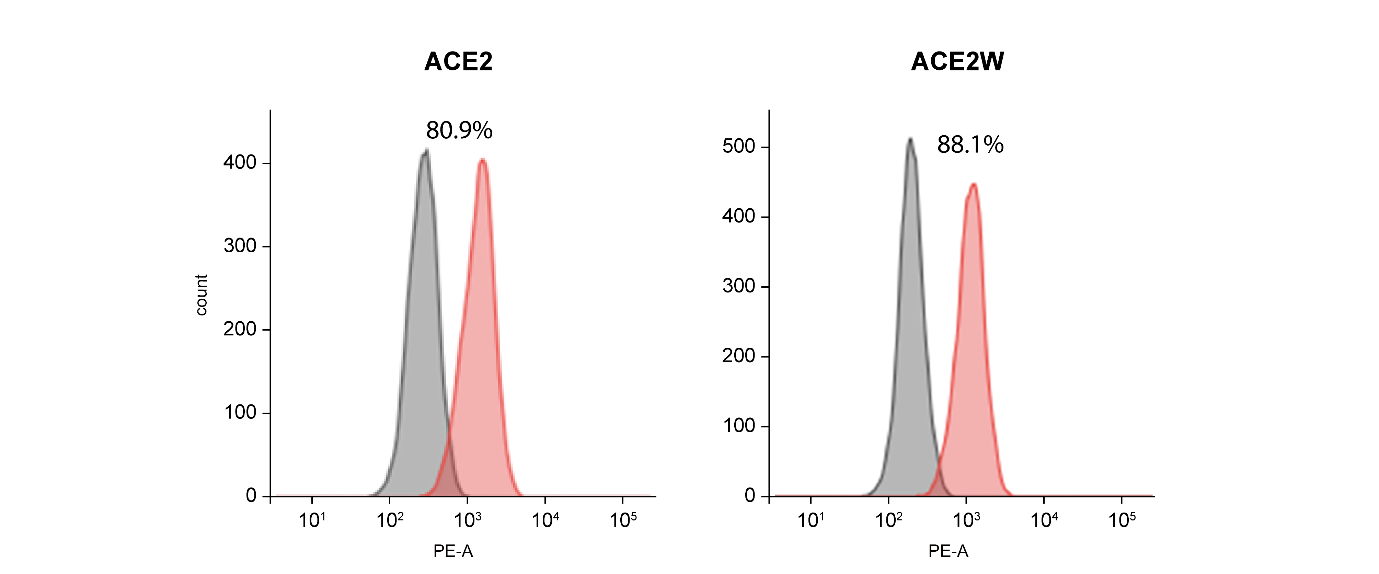


**Figure S5**. Flow cytometry for ATP1A1 expression in the wash-out and no–wash-out ACE2 populations at differentiation day 21.


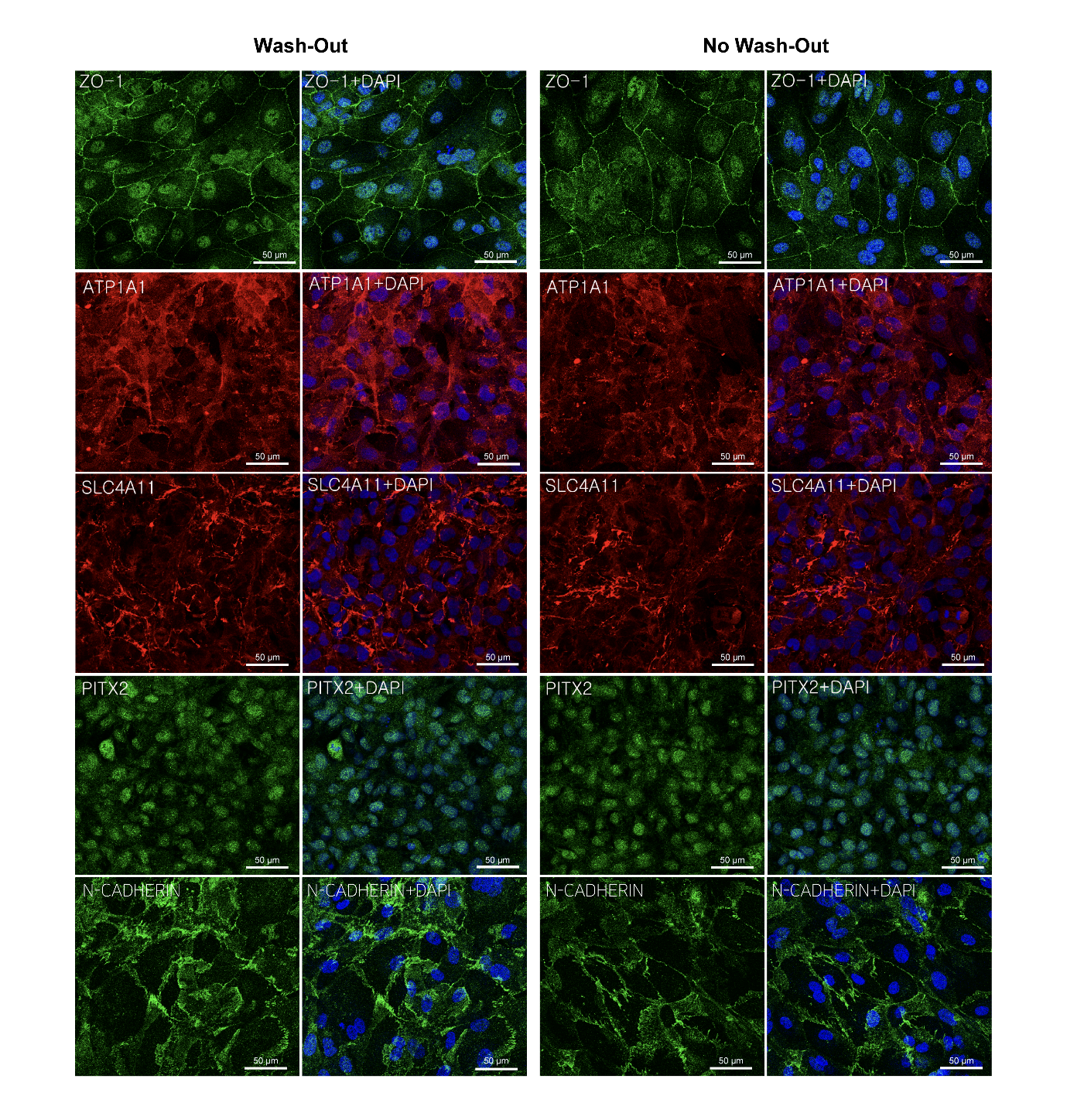


**Figure S6.** Immunocytochemistry (ICC) analysis of CEC markers in the wash-out and no–wash-out ACE2 populations at differentiation day 21.

**
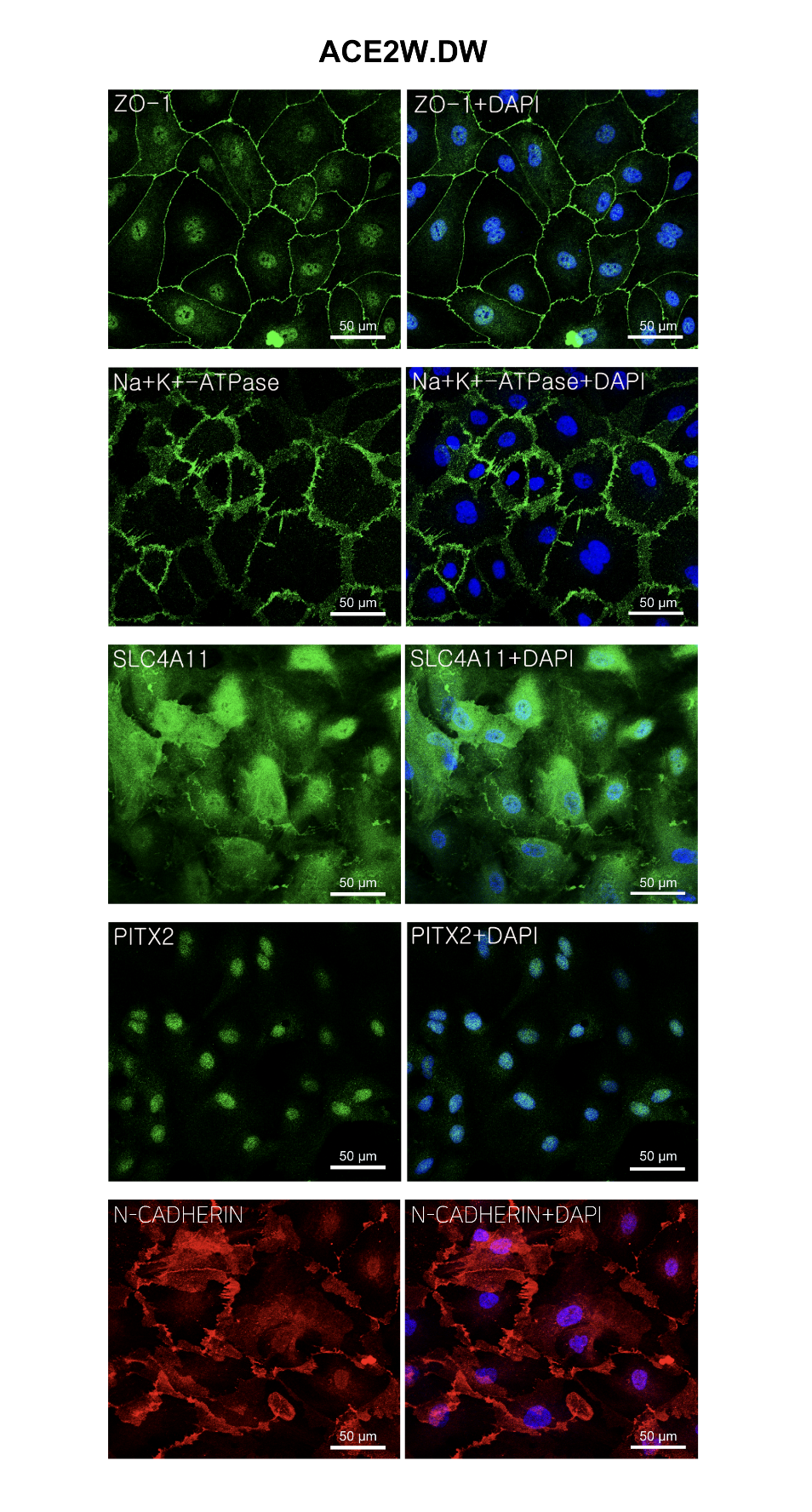
**

**Figure S7**. Immunocytochemistry (ICC) analysis of CEC markers in the ACE2W.DM population at differentiation day 14.


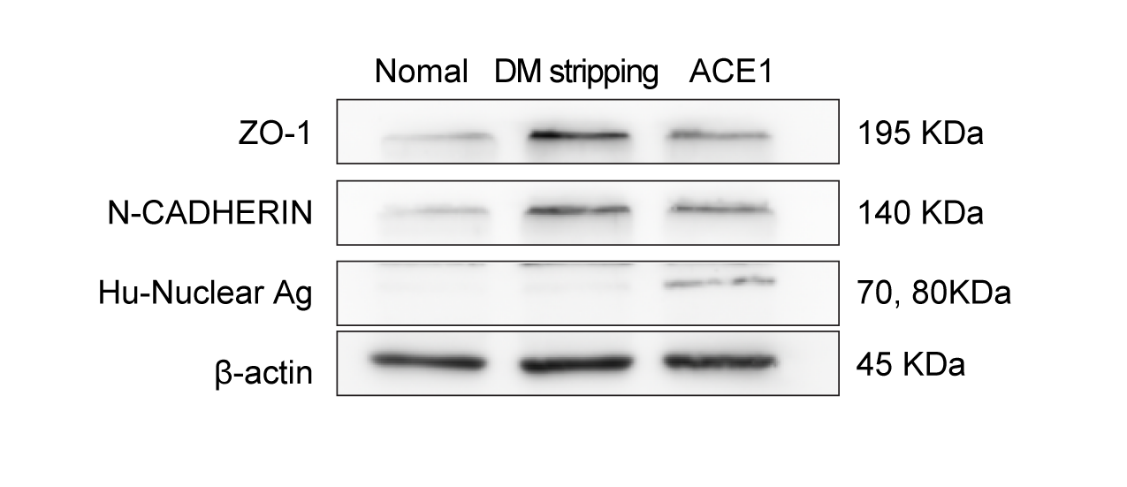


**Figure S8**. Western blot analysis of corneal tissue with ACE1 transplantation at 48 weeks post-transplant.


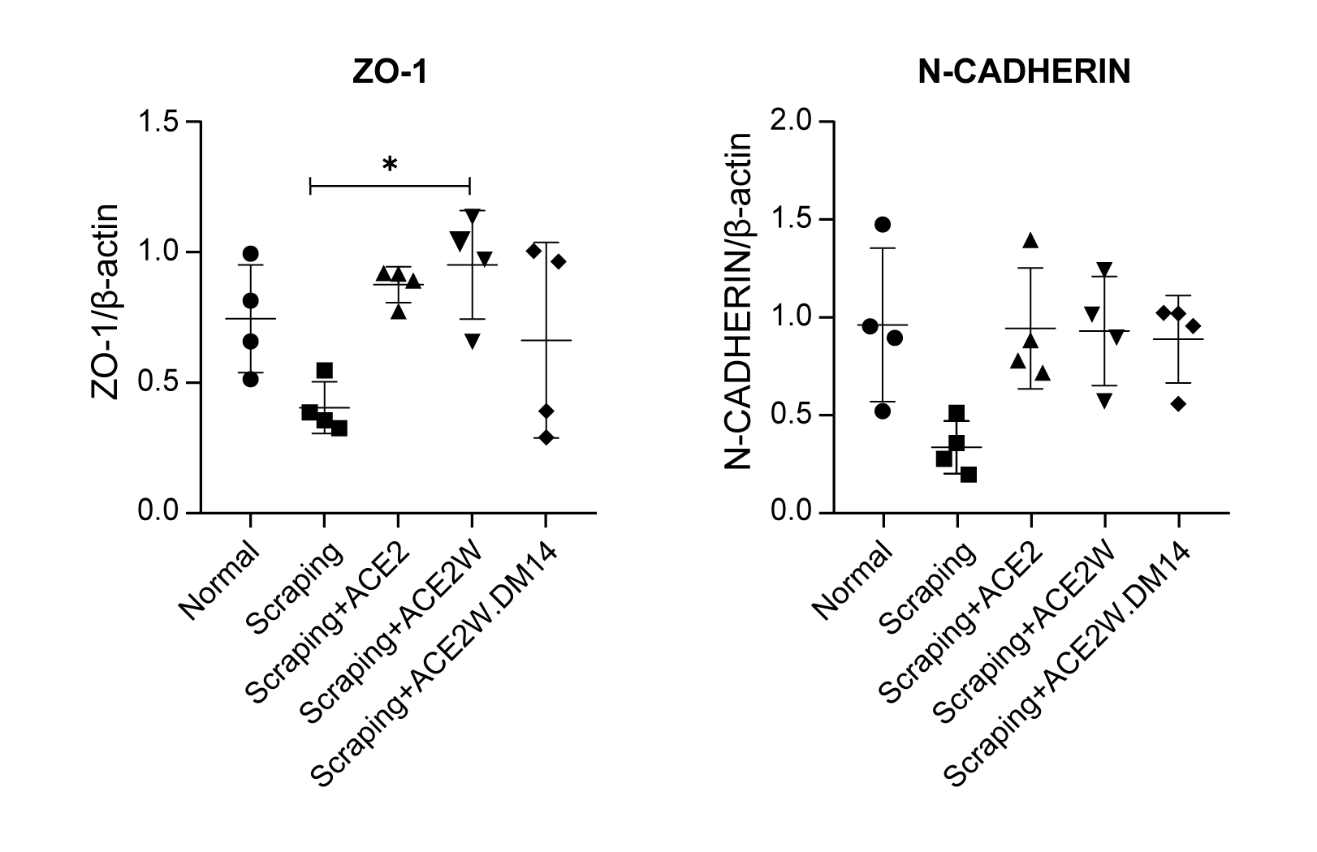


**Figure S9**. Western blot analysis of corneal tissue transplanted with different ACE2 populations at 2 weeks post-transplant. Quantitative data corresponding to Figure 6B are presented as graphs. (*p < 0.05)


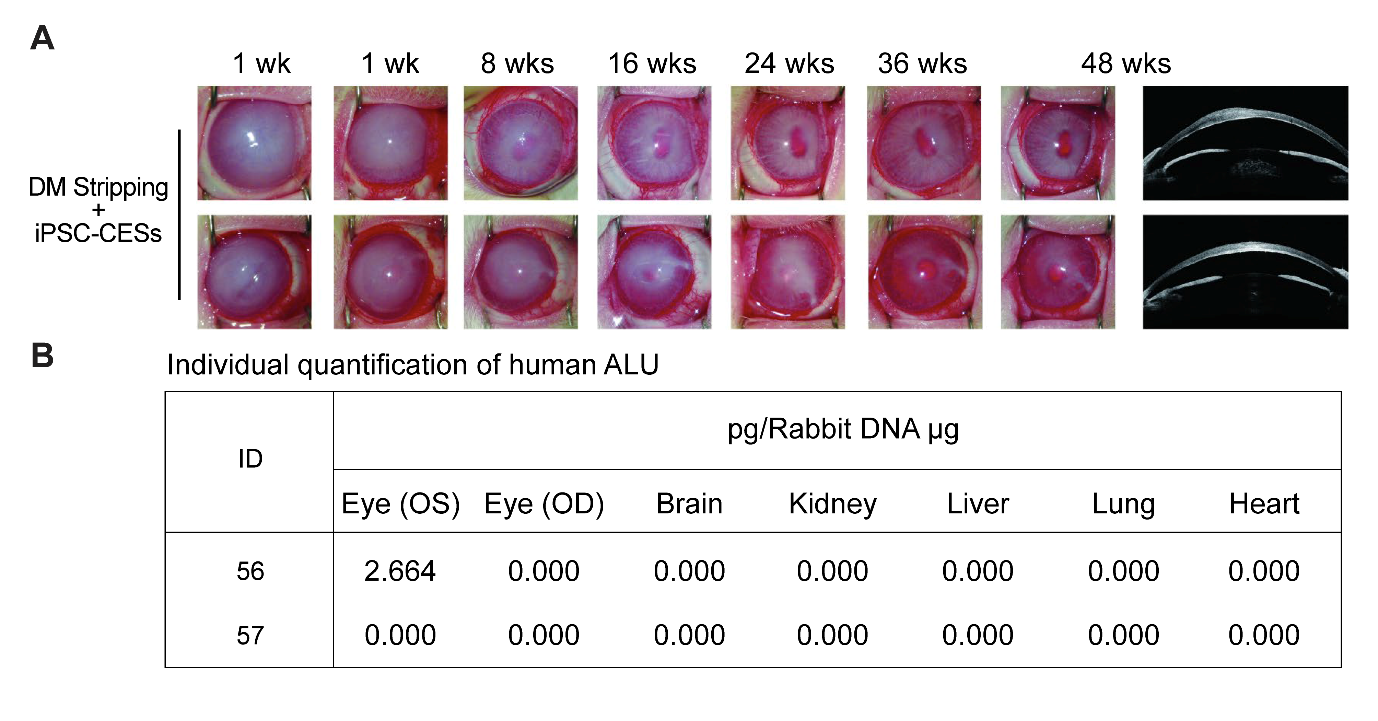


**Figure S10**. Biodistribution of transplanted iPSC-derived CECs in a rabbit CED model.

| REAGENT OR RESOURCE | SOURCE | IDENTIFIER |
| --- | --- | --- |
| Antibodies | | |
| ZO-1 | Invitrogen | Cat# 61-7300 |
| Sodium Potassium ATPase | Abcam | Cat# ab76020 |
| SLC4A11 | Novus biologicals | Cat# NBP1-46156 |
| N-CADHERIN | Cell signaling technology | Cat# 13116s |
| CD166 | BD Biosciences | Cat# 559260 |
| NCAM | R&D systems | Cat# MAB24081 |
| PITX2 | Abnova | Cat# H00005308-M01 |
| Human nuclear antigen | Abcam | Cat# ab191181 |
| STEM121 | Takara Bio | Cat# Y40410 |
| CD4 | Abcam | Cat# ab183685 |
| CD11b | Novus biologicals | Cat# NB110-89474 |
| CD8 | Abcam | Cat# ab228856 |
| β-actin | Cell signaling technology | Cat# 5125 |
| Goat anti-Mouse IgG (H+L) Secondary Antibody, HRP | Invitrogen | Cat# 31430 |
| Goat anti-Rabbit IgG (H+L) Secondary Antibody, HRP | Invitrogen | Cat# 65-6120 |
| PE Mouse Anti-Human CD166 | BD Biosciences | Cat# 559263 |
| PE mouse IgG1, κ Isotype control | BD Biosciences | Cat# 555749 |
| Alpha 1 Sodium Potassium ATPase/ATP1A1 Antibody (C464.6) | Santa Cruz Biotechnology | Cat# sc-21712 FITC |
| FITC Mouse IgG1, κ Isotype Control | BD Biosciences | Cat# 555748 |
| Alexa Fluor® 488 AffiniPure™ Donkey Anti-Mouse IgG (H+L) | Jackson immunoresearch | Cat# 715-545-150 |
| Alexa Fluor® 488 AffiniPure™ Donkey Anti-Rabbit IgG (H+L) | Jackson immunoresearch | Cat# 711-545-152 |
| Cy™3 AffiniPure™ Donkey Anti-Mouse IgG (H+L) | Jackson immunoresearch | Cat# 715-165-151 |
| Cy™3 AffiniPure™ Goat Anti-Rabbit IgG (H+L) | Jackson immunoresearch | Cat# 111-165-144 |
| Chemicals, peptides, and recombinant proteins | | |
| Vitronectin (VTN-N) Recombinant Human Protein, Truncated | Thermo fisher | Cat# A31804 |
| Fasudil | Stemcell technologies | Cat# 73662 |
| SB431542 | Selleckchem | Cat# s1067 |
| H 1152 dihydrochloride | Tocris | Cat# 2414 |
| L-Ascorbic acid 2-phosphate trisodium | MCE | Cat# HY-107837 |
| Insulin-Transferrin-Selenium (ITS -G) (100X) | Gibco | Cat# 41400045 |
| Human EGF, Recombinant Protein | Thermo fisher | Cat# AF-100-15-500UG |
| CaCl2 | Sigma-Aldrich | Cat# C7902 |
| TRIzol | Invitrogen | Cat# 15596018 |
| Critical commercial assays | | |
| Topscript cDNA synthesis kit | Enzynomics | Cat# EZ005M |
| PowerTrack SYBR Green Master mix | Applied Biosystems | Cat# A46109 |
| Experimental models: Cell lines | | |
| hiPSC | Yipcell Inc. | N/A |
| IHCE | Abm | RRID:CVCL_6E29 |
| Experimental models: Organisms/strains | | |
| New Zealand white rabbit | JA BIO (http://www.jabio.co.kr/index.php) | N/A |
| C57BL/6 mouse | JA BIO (http://www.jabio.co.kr/index.php) | RRID:MGI:2159769 |
| Primers | | |
| *GAPDH*_F (TCC AGA ACA TCA TCC CTG CC) | COSMO GENETECH | N/A |
| *GAPDH*_R (GCC TGC TTC ACC ACC TTC TT) | COSMO GENETECH | N/A |
| *CDH2*_F (GAC CAG GAC TAT GAC TTG AGC C) | COSMO GENETECH | N/A |
| *CDH2*_R (AGC TGT GGG GTC ATT GTC AG) | COSMO GENETECH | N/A |
| *COL8A1*_3F (GCC TCT CTC CCT GAT CTT ACG) | COSMO GENETECH | N/A |
| *COL8A1*_3R (GCA GCA CAG CCA TCA CAT TT) | COSMO GENETECH | N/A |
| *POU5F1*_F (TTT TGG TAC CCC AGG CTA TG) | COSMO GENETECH | N/A |
| *POU5F1*_R (GCA GGC ACC TCA GTT TGA AT) | COSMO GENETECH | N/A |
| *NANOG*_F (ACC TTG GCT GCC GTC TCT GG) | COSMO GENETECH | N/A |
| *NANOG*_R (AGC AAA GCC TCC CAA TCC CAA ACA) | COSMO GENETECH | N/A |
| *PODXL*_F (AAC TGG GCA AAG TGT GAG GA) | COSMO GENETECH | N/A |
| *PODXL*_R (ACT TAT CTT GGG CCG GGT TG) | COSMO GENETECH | N/A |
| Human mito 3F (GCC TTC CCC CGT AAA TGA TA) | COSMO GENETECH | N/A |
| Human mito 3R (CTT CTG TGG AAC GAG GGT TT) | COSMO GENETECH | N/A |
| Human mito 5F (GCC GAC CGT TGA CTA TTC TC) | COSMO GENETECH | N/A |
| Human mito 5R (GGG GGC ATC CAT ATA GTC AC) | COSMO GENETECH | N/A |
| Software and algorithms | | |
| GraphPad Prism | GraphPad Software, LLC (www.graphpad.com) | RRID:SCR_002798 |
| FlowJo | FlowJo, LLC (www.flowjo.com) | RRID:SCR_008520 |
| Other | | |
| Human donor cornea | Eversight | N/A |

**Table S1**. Key resources table
